# Supplementary material for: Exploring Pyrrolo-Fused Heterocycles as Promising Anticancer Agents: An Integrated Synthetic, Biological, and Computational Approach
Source: Pharmaceuticals (Basel). 2023 Jun 11;16(6):865. doi: 10.3390/ph16060865 (PMC10305032; doi:10.3390/ph16060865)
Supplement: Supplementary file 1 [file pharmaceuticals-16-00865-s001.zip › pharmaceuticals-2363081-supplementary.pdf]

Supporting Information
for

Exploring Pyrrolo-Fused Heterocycles as Promising Anticancer Agents: An Integrated Synthetic, Biological and Computational Approach

Roxana-Maria Amărandi [1], Maria-Cristina Al-Matarneh [2,3,*], Lăcrămioara Popovici [3], Catalina Ionica Ciobanu [4], Andrei Neamțu [1], Ionel I. Mangalagiu [3] and Ramona Danac [3,*]

[1]TRANSCEND Research Center, Regional Institute of Oncology, 2-4 General Henri Mathias Berthelot Street, Iași 700483, Romania; rpomohaci@iroiasi.ro (R.M.A.); neamtuandrei@gmail.com (A.N.)
[2]"Petru Poni" Institute of Macromolecular Chemistry of Romanian Academy, 41A Grigore Ghica Voda Alley, Iasi 700487, Romania;
[3]Faculty of Chemistry, Alexandru Ioan Cuza University of Iasi, 11 Carol I, Iasi 700506, Romania; tiru.lacramioara@yahoo.com (L.P.); ionelm@uaic.ro (I.I.M.)
[4]Institute of Interdisciplinary Research-CERNESIM Centre, Alexandru Ioan Cuza University of Iasi, 11 Carol I, Iasi 700506, Romania; catalina.ciobanu@uaic.ro (C.I.C)
*Correspondence: rdanac@uaic.ro (R.D.); almatarneh.cristina@icmpp.ro (M.C.A.M.)

Table of Contents

Figure S1. HR-MS spectrum of compound **10a**……………………………………………………4
Figure S2. ^{1}H-NMR spectrum of compound **10a**……………………………………………4
Figure S3. ^{13}C-NMR spectrum of compound **10a**……………………………………………5
Figure S4. ^{1}H-NMR spectrum of compound **10b**……………………………………………5
Figure S5. ^{13}C-NMR spectrum of compound **10b**……………………………………………6
Figure S6. ^{1}H-NMR spectrum of compound **10c**……………………………………………6
Figure S7. ^{13}C-NMR spectrum of compound **10c**……………………………………………7
Figure S8. ^{1}H-NMR spectrum of compound **12a**……………………………………………7
Figure S9. ^{13}C-NMR spectrum of compound **12a**……………………………………………8
Figure S10. ^{1}H-NMR spectrum of compound **12b**……………………………8
Figure S11. ^{13}C-NMR spectrum of compound **12b**……………………………9
Figure S12. ^{1}H-NMR spectrum of compound **12c**……………………………9
Figure S13. ^{13}C-NMR spectrum of compound **12c**……………………………10
Figure S14. ^{1}H-NMR spectrum of compound **12d**……………………………10
Figure S15. ^{13}C-NMR spectrum of compound **12d**……………………………11
Figure S16. ^{1}H-NMR spectrum of compound **13b**……………………………11
Figure S17. ^{13}C-NMR spectrum of compound **13b**……………………………12
Figure S18. ^{1}H-NMR spectrum of compound **16b**……………………………12
Figure S19. ^{13}C-NMR spectrum of compound **16**……………………………13
Figure S20. Results of the *in vitro* growth of cancer cell lines in the single-dose assay for compound **4b**….14

Figure S21. Results of the *in vitro* growth of cancer cell lines in the single-dose assay for compound **4c**….15
Figure S22. Results of the *in vitro* growth of cancer cell lines in the single-dose assay for compound **4d**….16
Figure S23. Results of the *in vitro* growth of cancer cell lines in the single-dose assay for compound **7b**….17
Figure S24. Results of the *in vitro* growth of cancer cell lines in the single-dose assay for compound **7c**….18
Figure S25. Results of the *in vitro* growth of cancer cell lines in the single-dose assay for compound **9a**….19
Figure S26. Results of the *in vitro* growth of cancer cell lines in the single-dose assay for compound **9d**….20
Figure S27. Results of the *in vitro* growth of cancer cell lines in the single-dose assay for compound **10a**...21
Figure S28. Results of the *in vitro* growth of cancer cell lines in the single-dose assay for **10b**……………22
Figure S29. Results of the *in vitro* growth of cancer cell lines in the single-dose assay for **10c**……………23
Figure S30. Results of the *in vitro* growth of cancer cell lines in the single-dose assay for **10d**……………24
Figure S31. Results of the *in vitro* growth of cancer cell lines in the single-dose assay for **12a**……………25
Figure S32. Results of the *in vitro* growth of cancer cell lines in the single-dose assay for **12b**……………26
Figure S33. Results of the *in vitro* growth of cancer cell lines in the single-dose assay for **12c**……………27
Figure S34. Results of the *in vitro* growth of cancer cell lines in the single-dose assay for **12d**……………28
Figure S35. Results of the *in vitro* growth of cancer cell lines in the single-dose assay for **13a**……………29
Figure S36. Results of the *in vitro* growth of cancer cell lines in the single-dose assay for **13b**……………30
Figure S37. Results of the *in vitro* growth of cancer cell lines in the single-dose assay for **13c**……………31
Figure S38. Results of the *in vitro* growth of cancer cell lines in the single-dose assay for **13d**……………32
Figure S39. Results of the *in vitro* growth of cancer cell lines in the single-dose assay for **15a**……………33
Figure S40. Results of the *in vitro* growth of cancer cell lines in the single-dose assay for **15d**……………34
Figure S41. Results of the *in vitro* growth of cancer cell lines in the single-dose assay for **16a**……………35
Figure S42. Results of the *in vitro* growth of cancer cell lines in the single-dose assay for **16b**……………36
Figure S43. Results of the *in vitro* growth of cancer cell lines in the single-dose assay for **16c**……………37
Figure S44. Results of the *in vitro* growth of cancer cell lines in the single-dose assay for **16d**……………38
Figure S45. Results of the 5-dose *in vitro* human cancer cell growth inhibition for **10a** …………………...…39
Figure S46. Conformation distribution after blind docking of compounds (a) colchicine (b) Phen; (c) **10a**...40
Figure S47. Binding score distribution and RMSD from lowest-scoring solution from docking experiments……………………………………………………………………………………………41
Figure S48. Local docking to α,β-tubulin and superimposition with colchicine for (a,d) BM I; (b,e) BM II; (c,f) BM III………………………………………………………………………………………....42
Figure S49. Superimposition of BM II of **10a** from local docking and D64131 (PDB ID 6K9V) …………43
Figure S50. Superimposition of BM I of **10a** from local docking and the last frame of the MD simulation..43
Figure S51. Ligand Root Mean Square Fluctuation (RMSF) throughout the simulations for (A) **10a** and (B) Colchicine...44
Figure S52. Timeline representation of number of H-bond interactions throughout the MD simulations for (A) BM I; (B) BM II; (C) BM III; (D) Colchicine………………………………………………………….…44

Figure S53. Timeline representation of number of polar interactions throughout the MD simulations for (A) BM I; (B) BM II; (C) BM III; (D) Colchicine……………………………………………………………...45

Figure S54. Timeline representation of number of all interactions throughout the MD simulations for (A) BM I; (B) BM II; (C) BM III; (D) Colchicine……………………………………………………………...45

Figure S55. Configurational entropy for free **10a**, and BM I, BM II and BM III when bound to tubulin, and the difference between free and bound state……………………………………………………..…...46

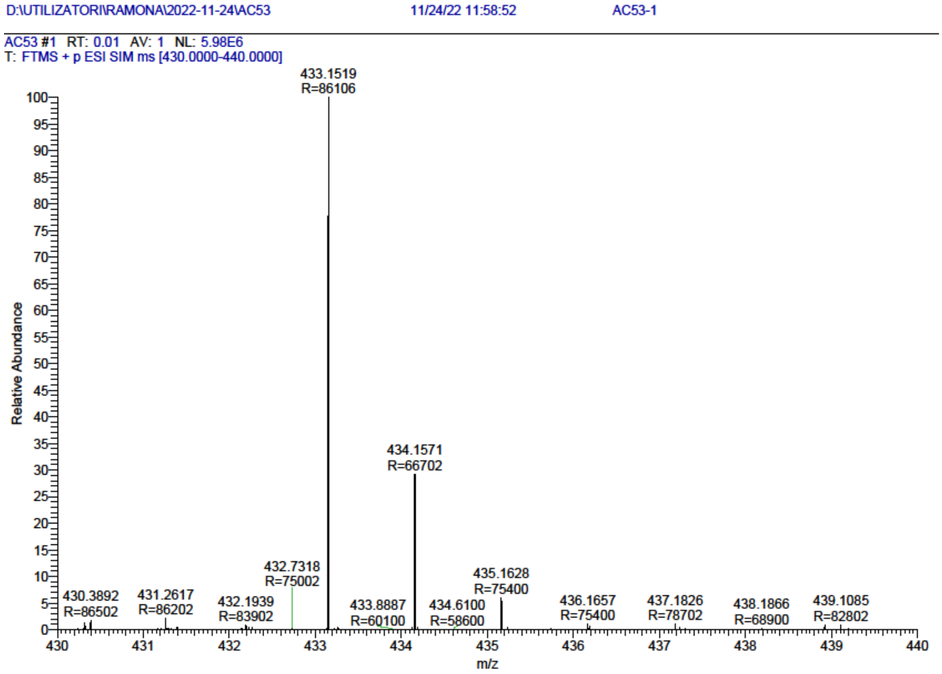

Figure S1. HR-MS spectrum of compound **10a**

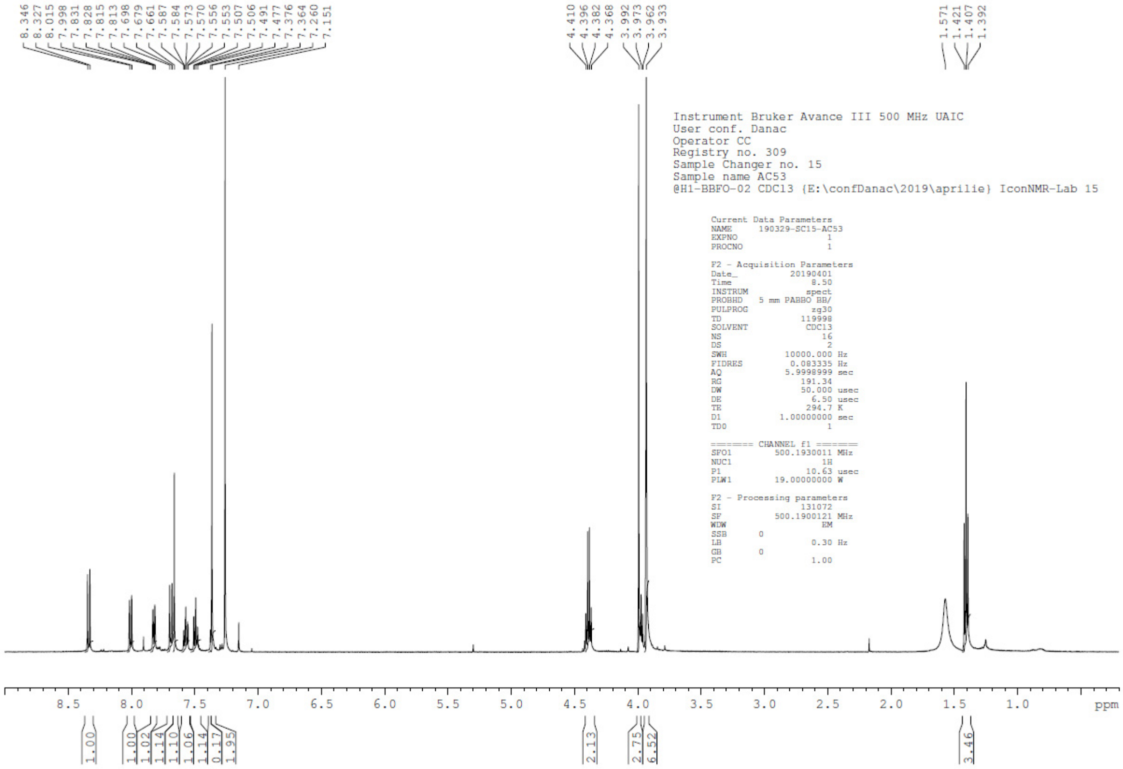

Figure S2. ^{1}H-NMR spectrum of compound **10a**

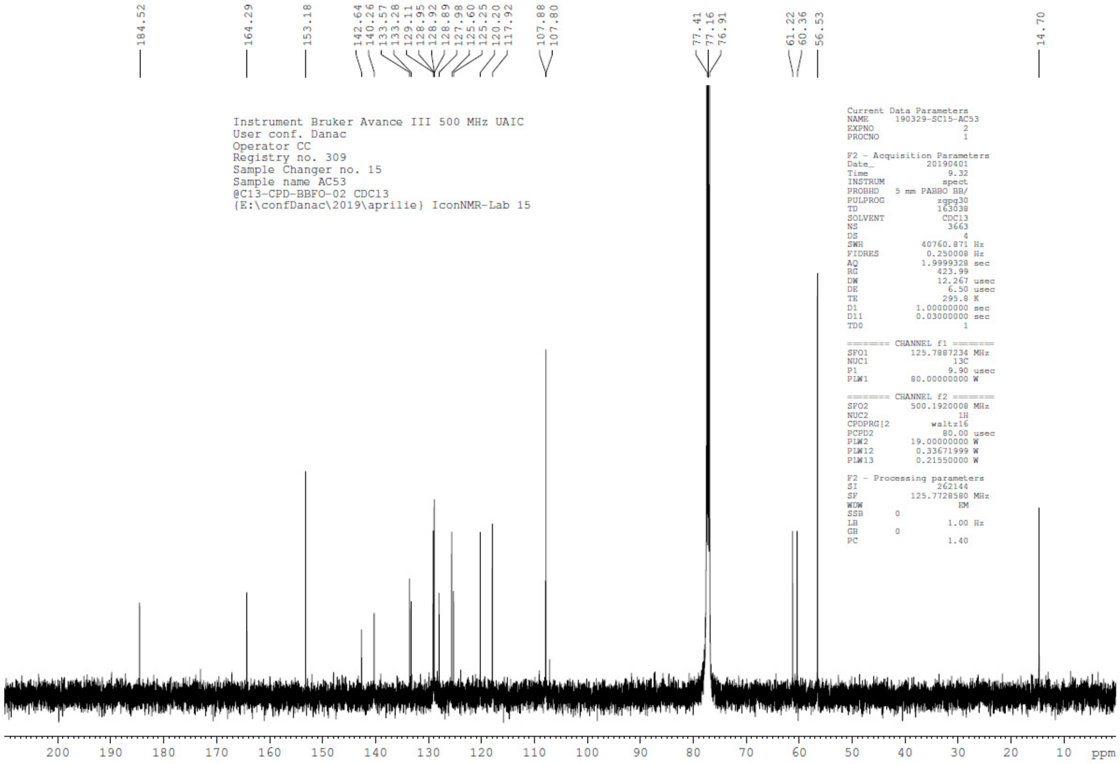

Figure S3. ^{13}C-NMR spectrum of compound **10a**

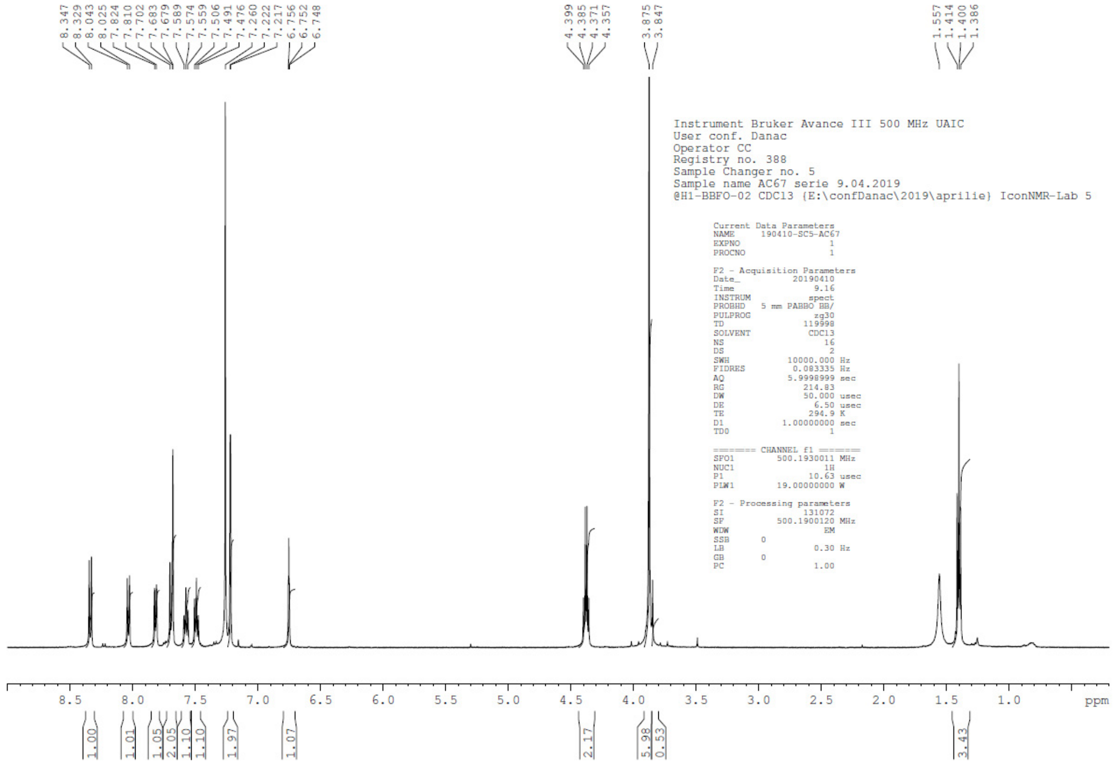

Figure S4. ^{1}H-NMR spectrum of compound **10b**

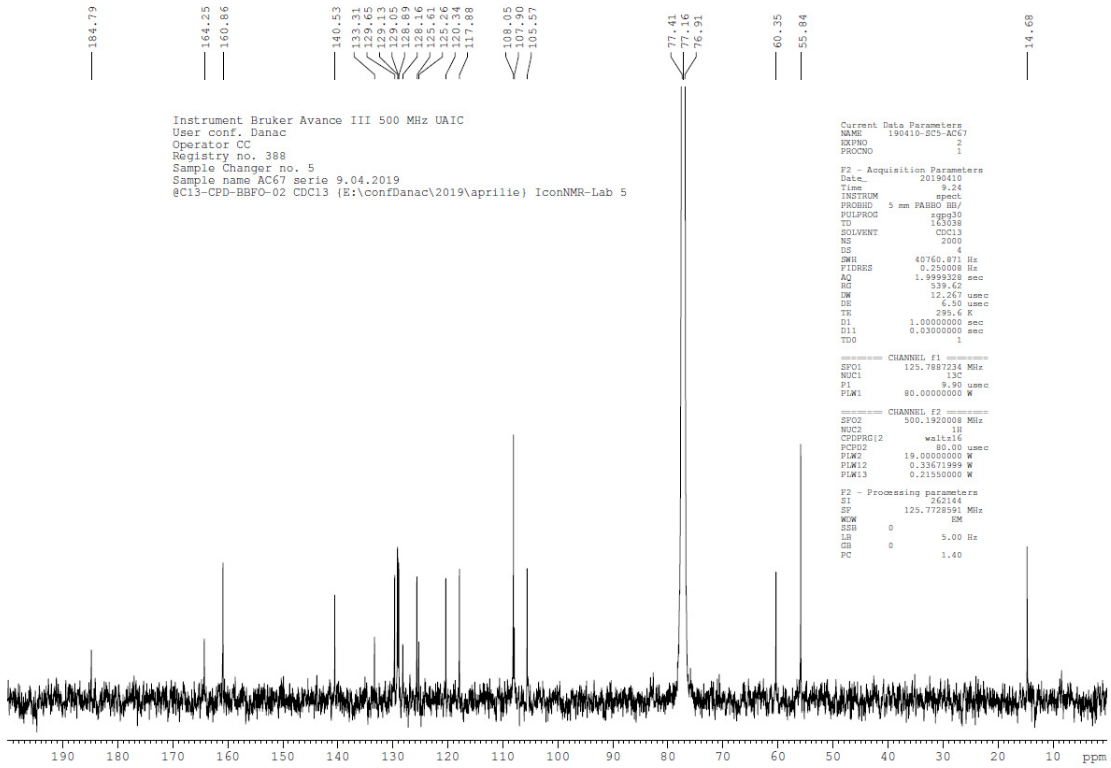

Figure S5. ^{13}C-NMR spectrum of compound **10b**

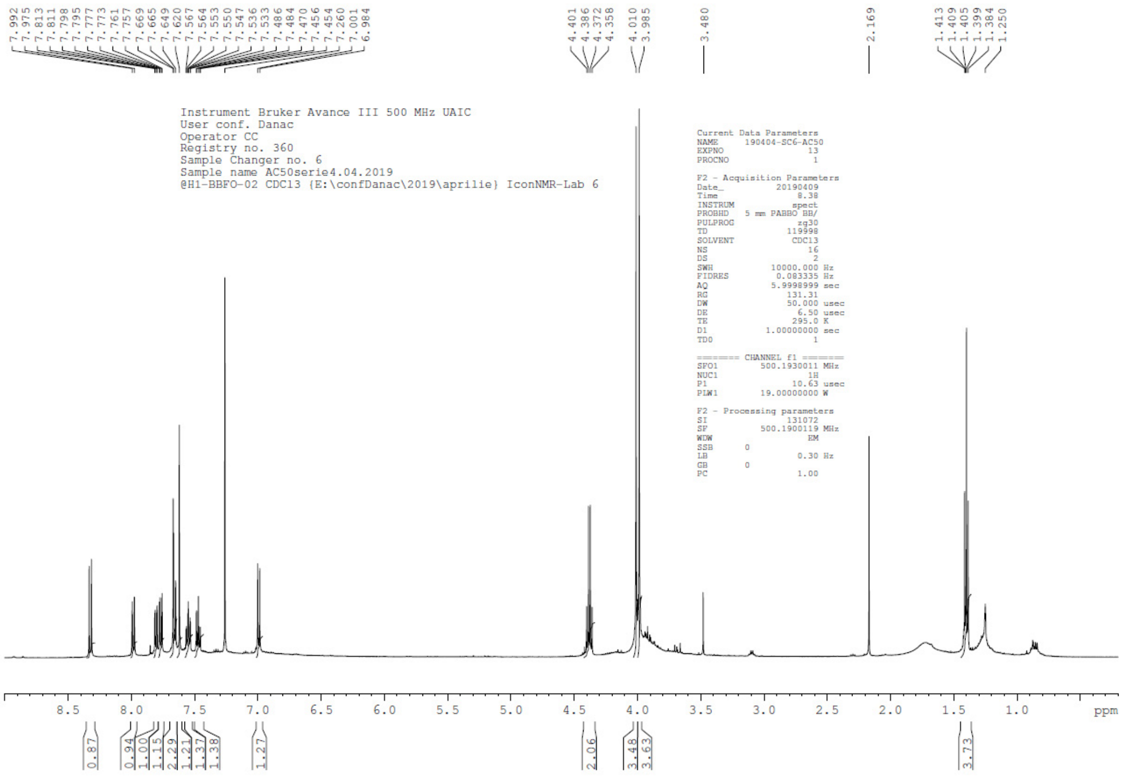

Figure S6. ^{1}H-NMR spectrum of compound **10c**

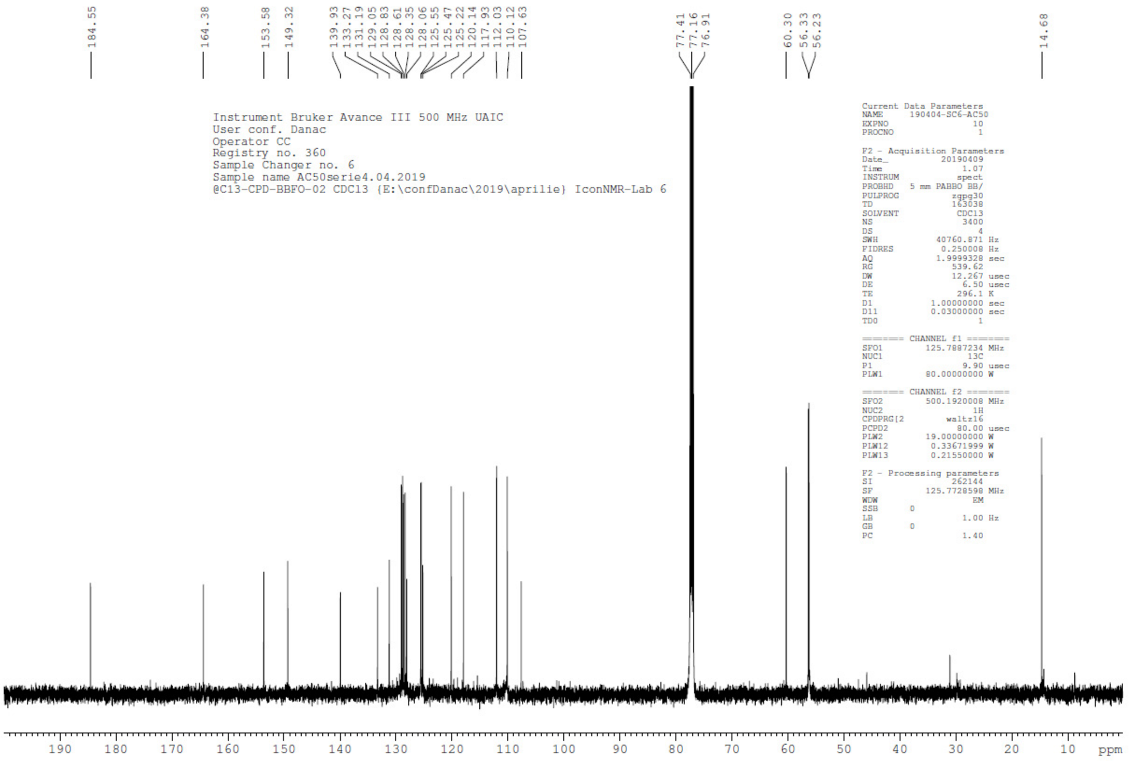

Figure S7. ^{13}C-NMR spectrum of compound **10c**

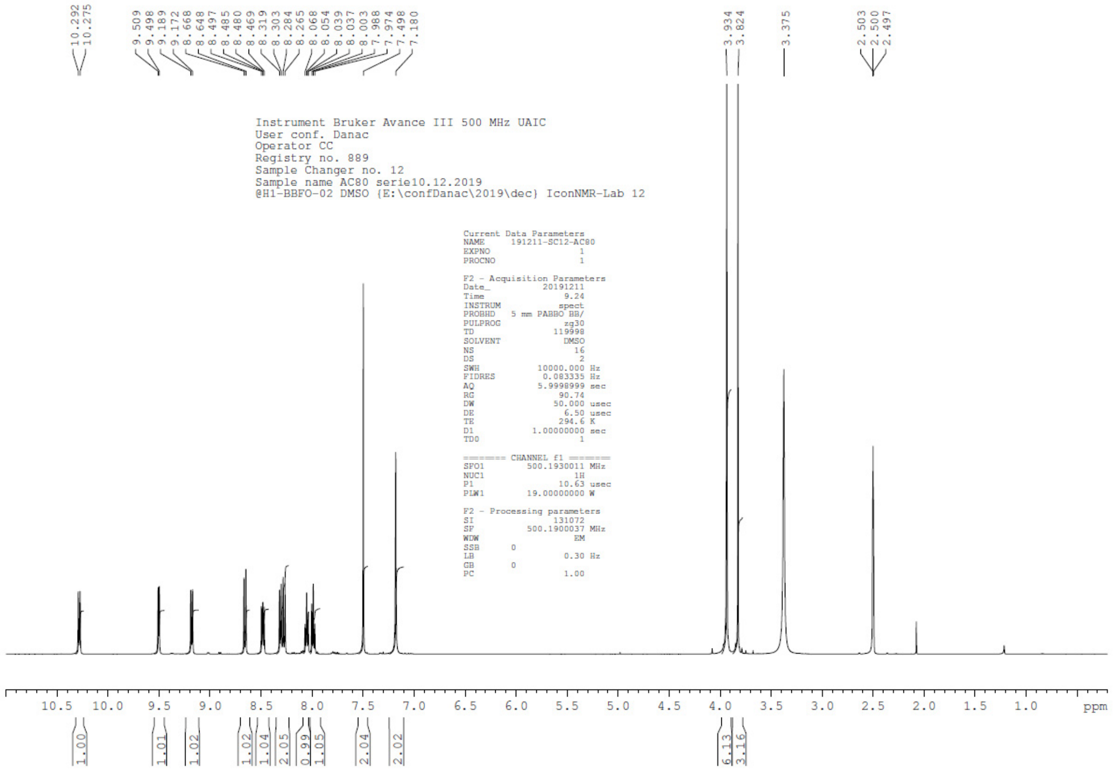

Figure S8. ^{1}H-NMR spectrum of compound **12a**

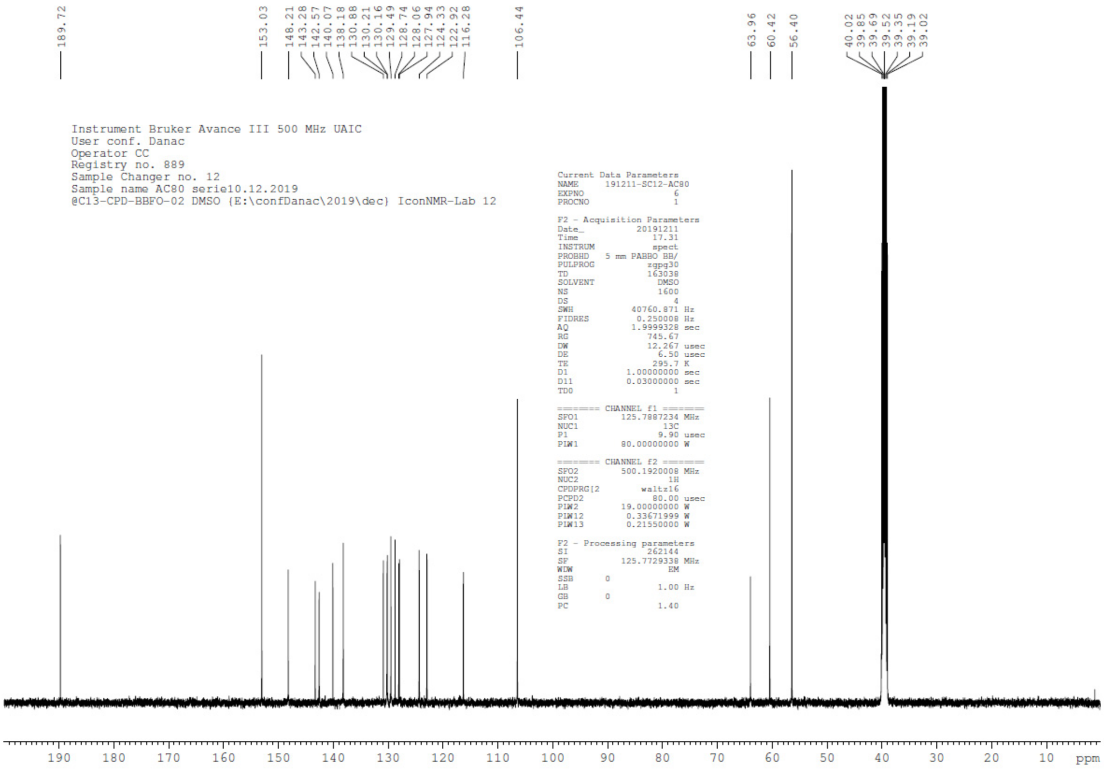

Figure S9. ^{13}C-NMR spectrum of compound **12a**

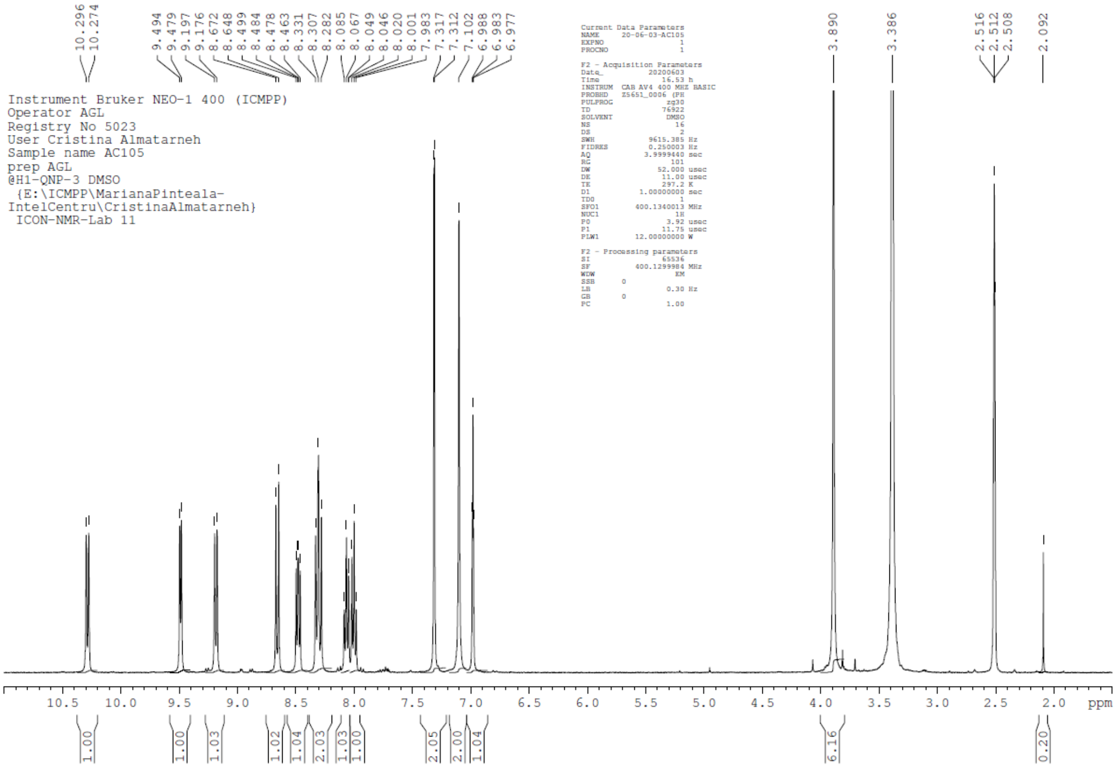

Figure S10. ^{1}H-NMR spectrum of compound **12b**

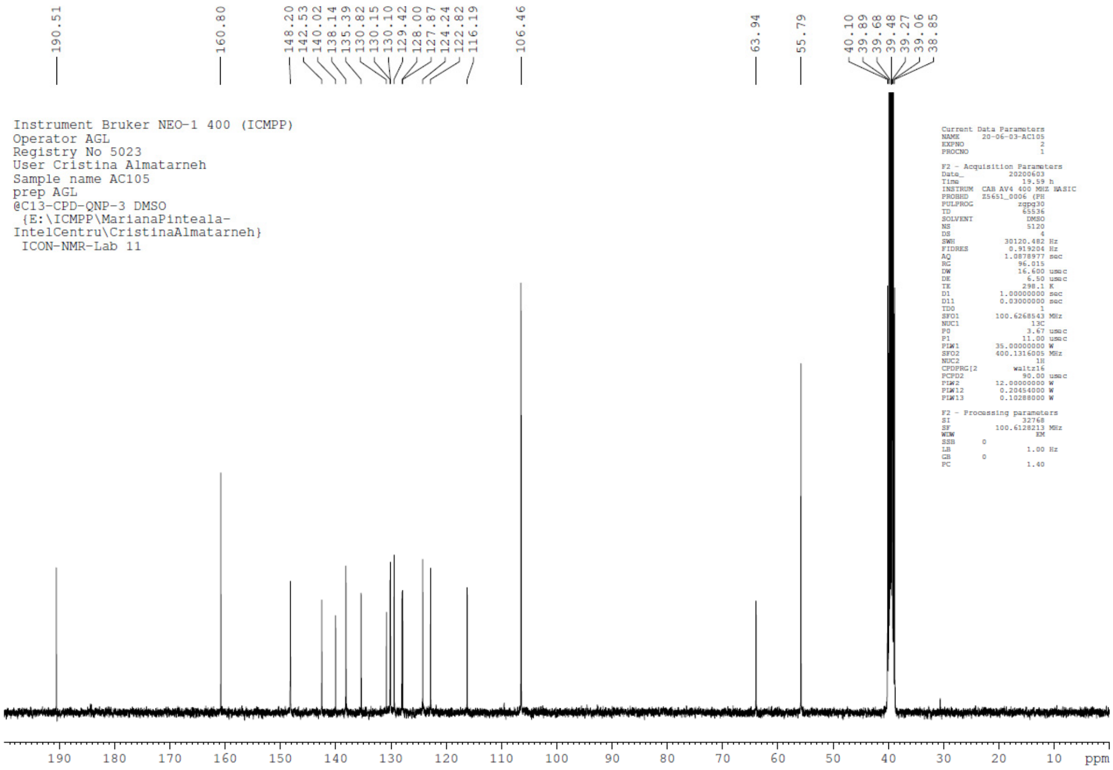

Figure S11. ^{13}C-NMR spectrum of compound **12b**

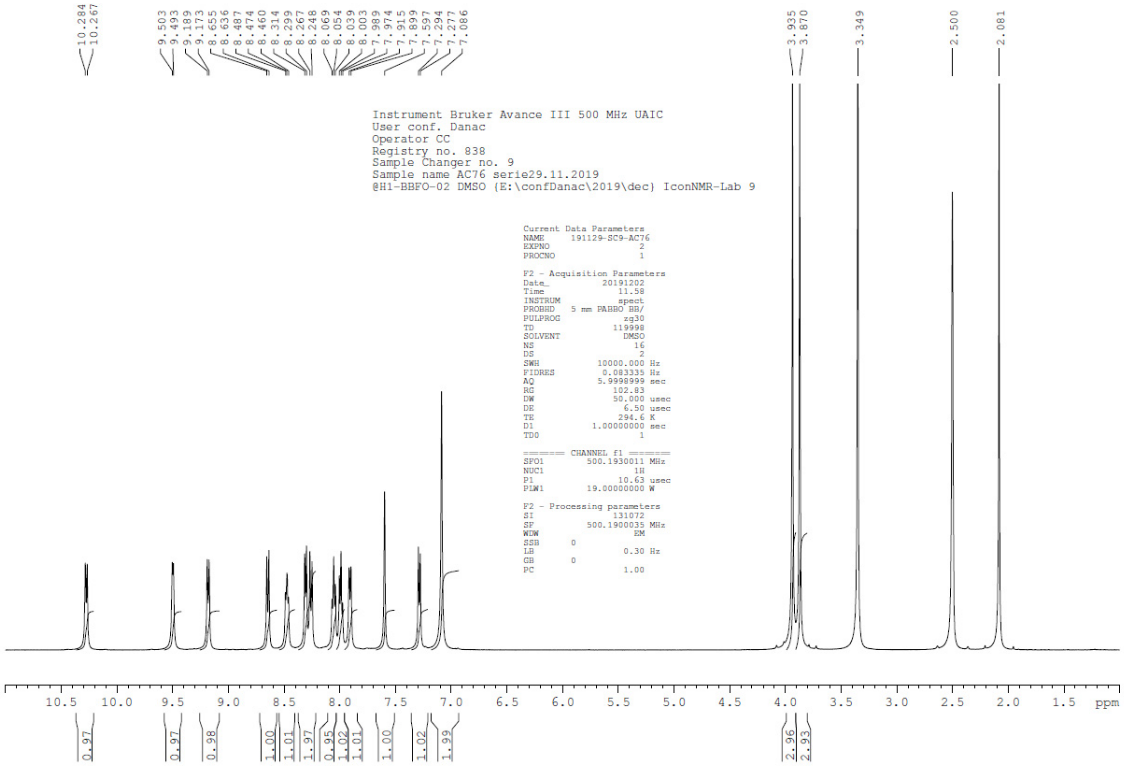

Figure S12. ^{1}H-NMR spectrum of compound **12c**

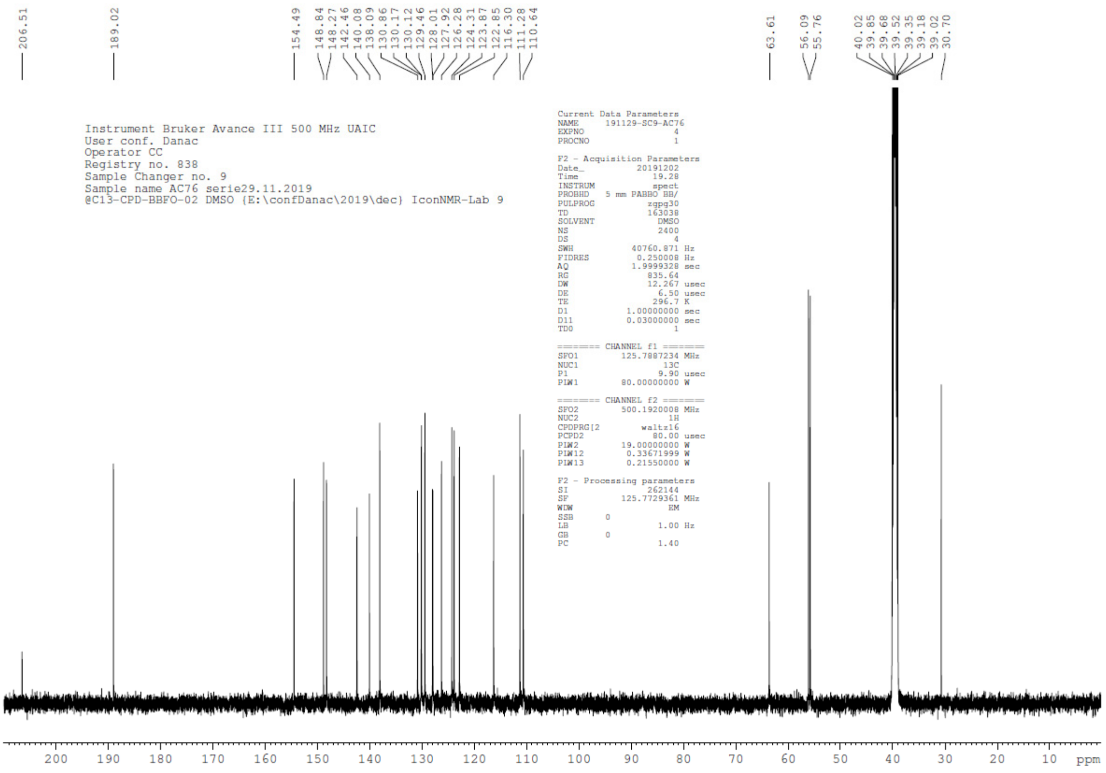

Figure S13. ^{13}C-NMR spectrum of compound **12c**

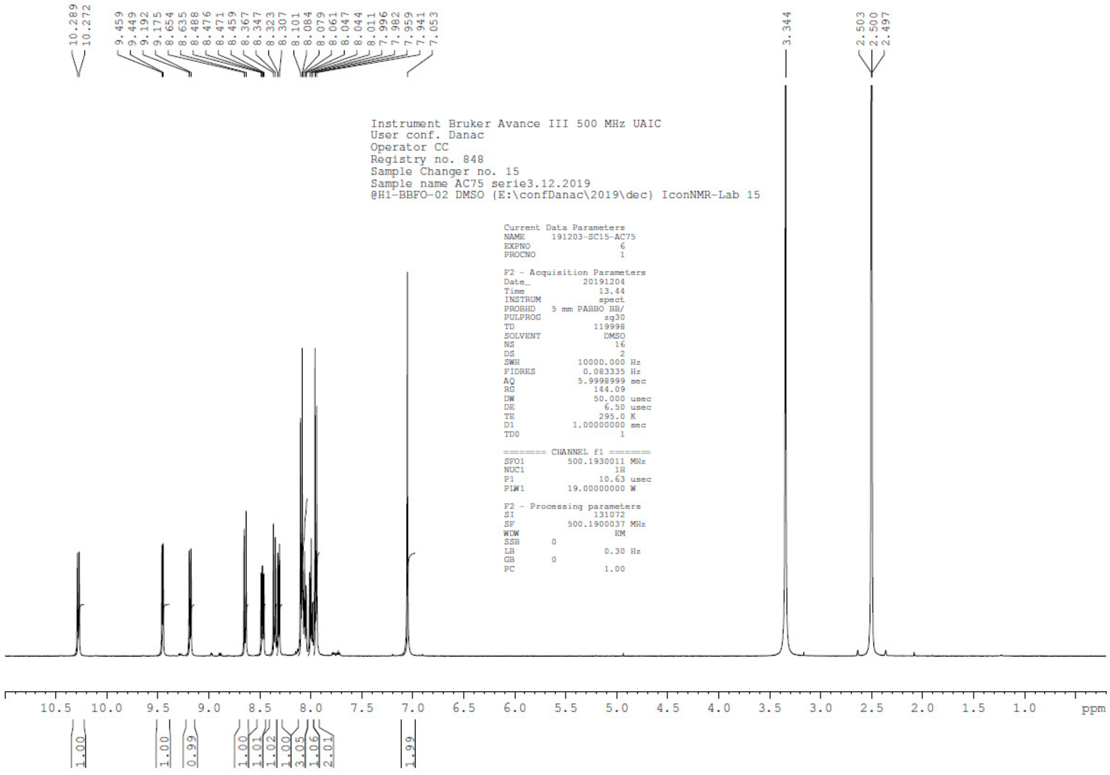

Figure S14. ^{1}H-NMR spectrum of compound **12d**

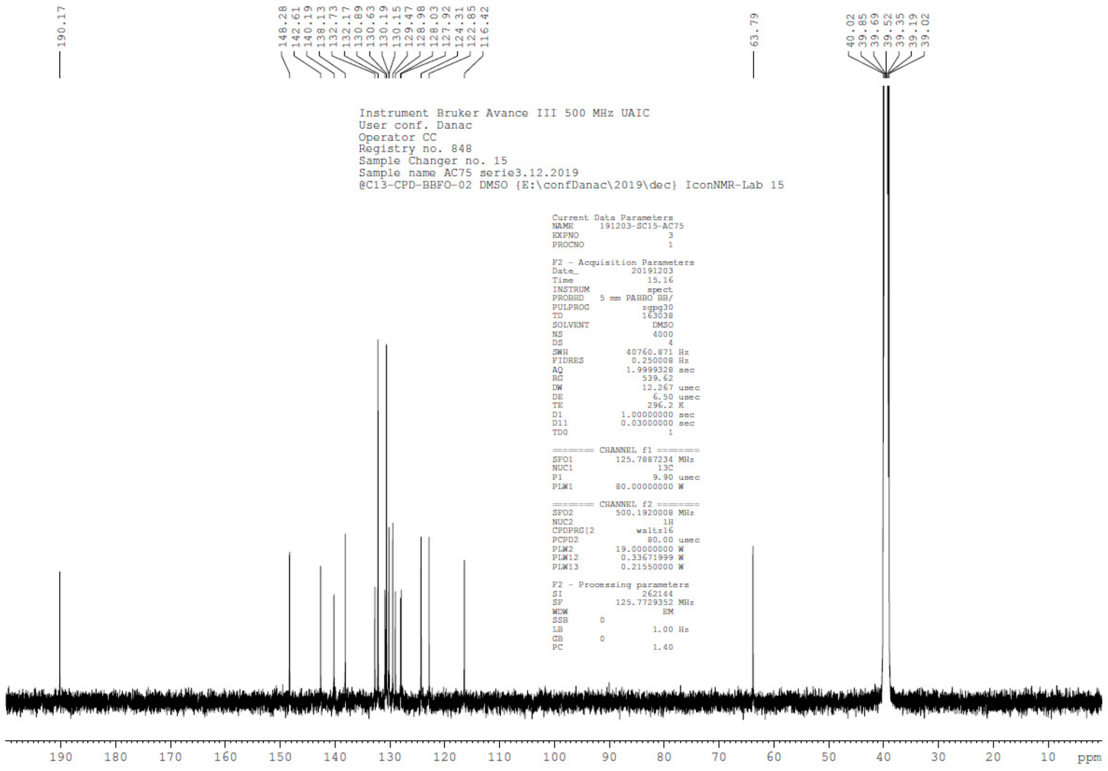

Figure S15. ^{13}C-NMR spectrum of compound **12d**

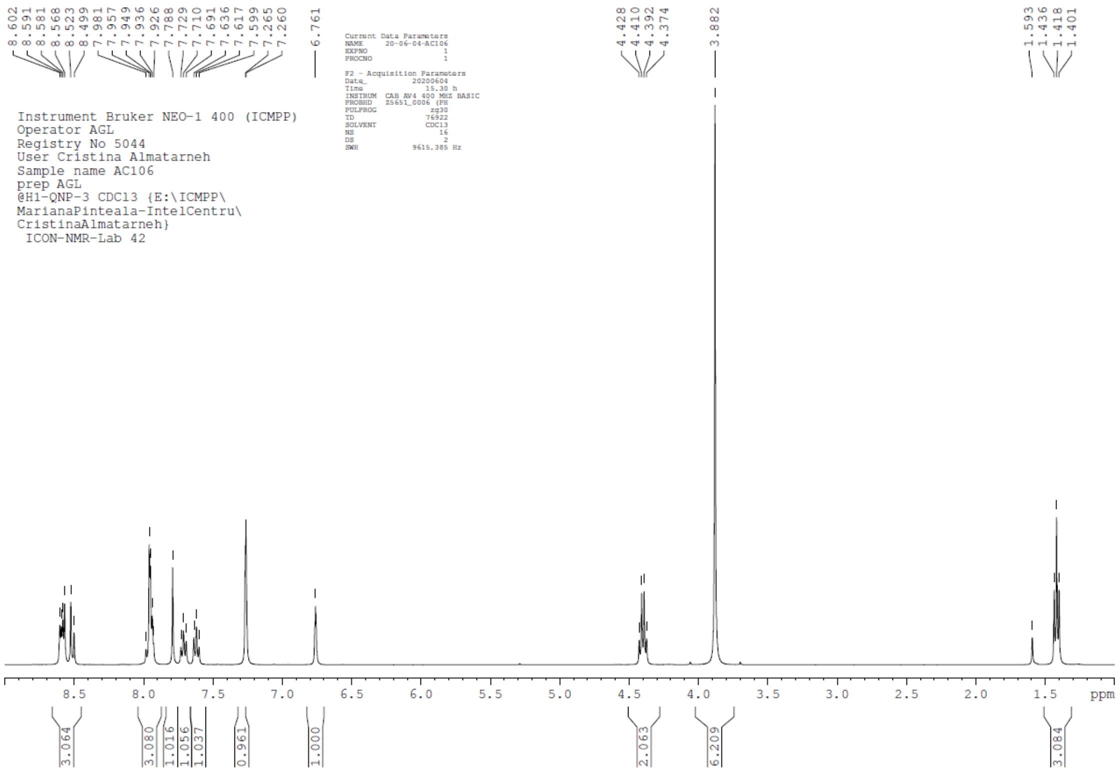

Figure S16. ^{1}H-NMR spectrum of compound **13b**

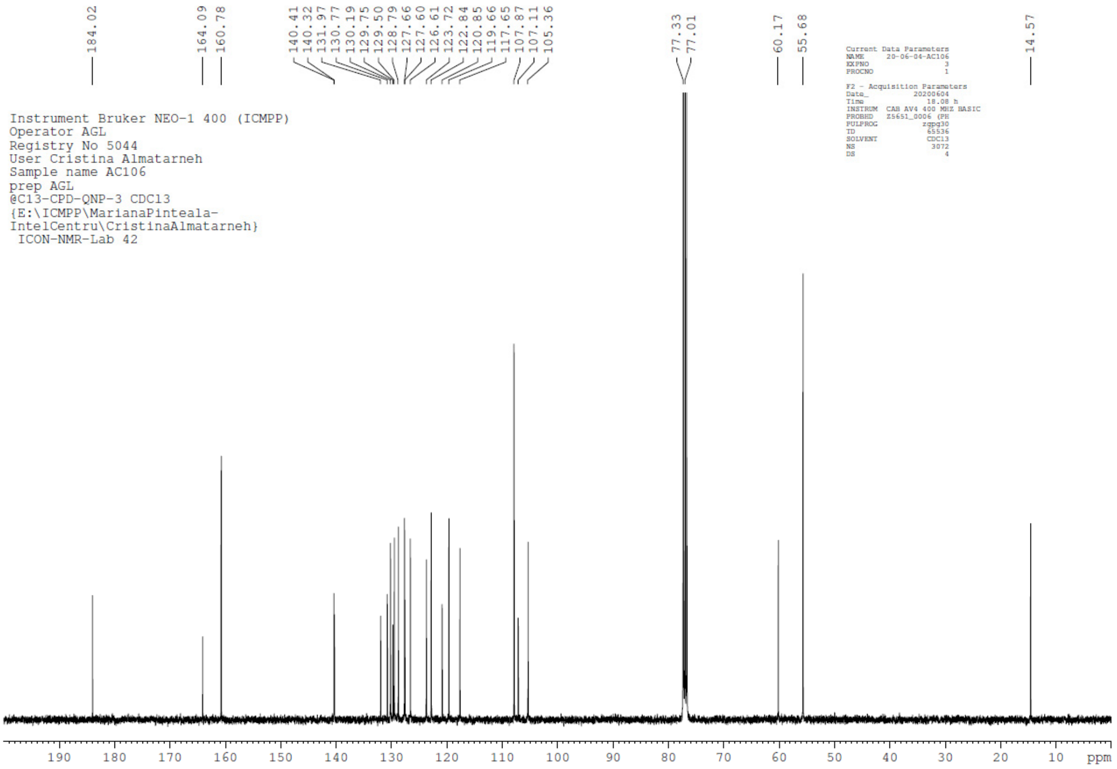

Figure S17. ^{13}C-NMR spectrum of compound **13b**

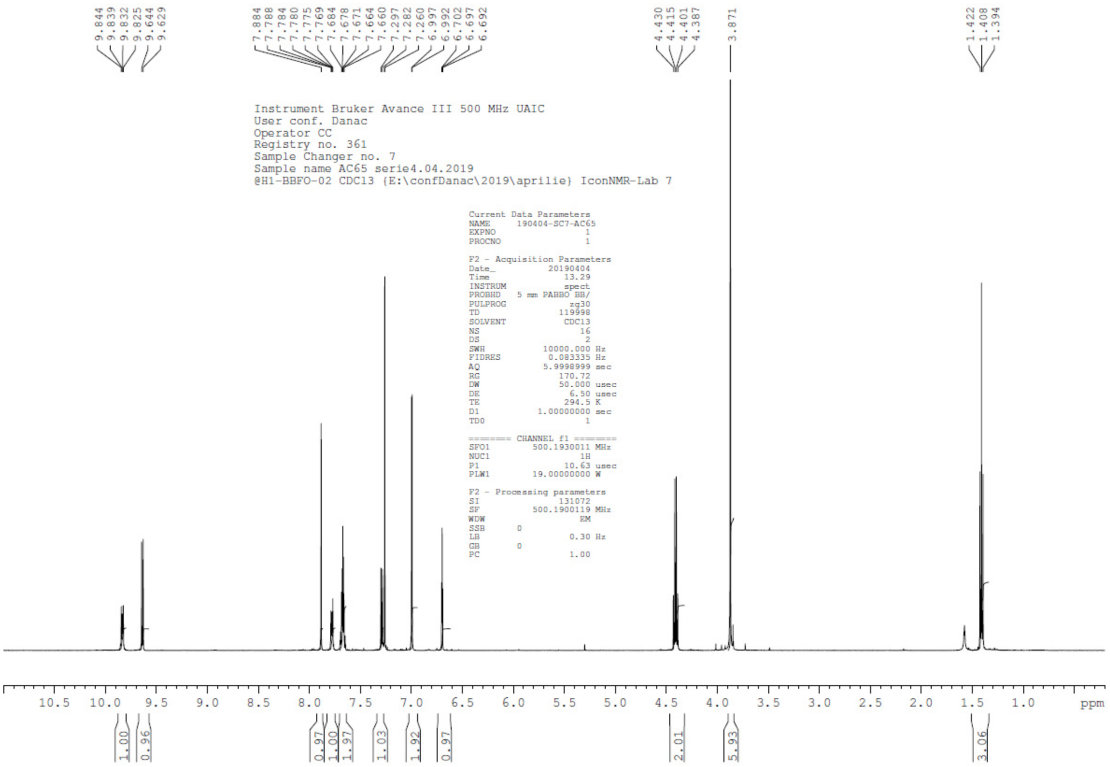

Figure S18. ^{1}H-NMR spectrum of compound **16b**

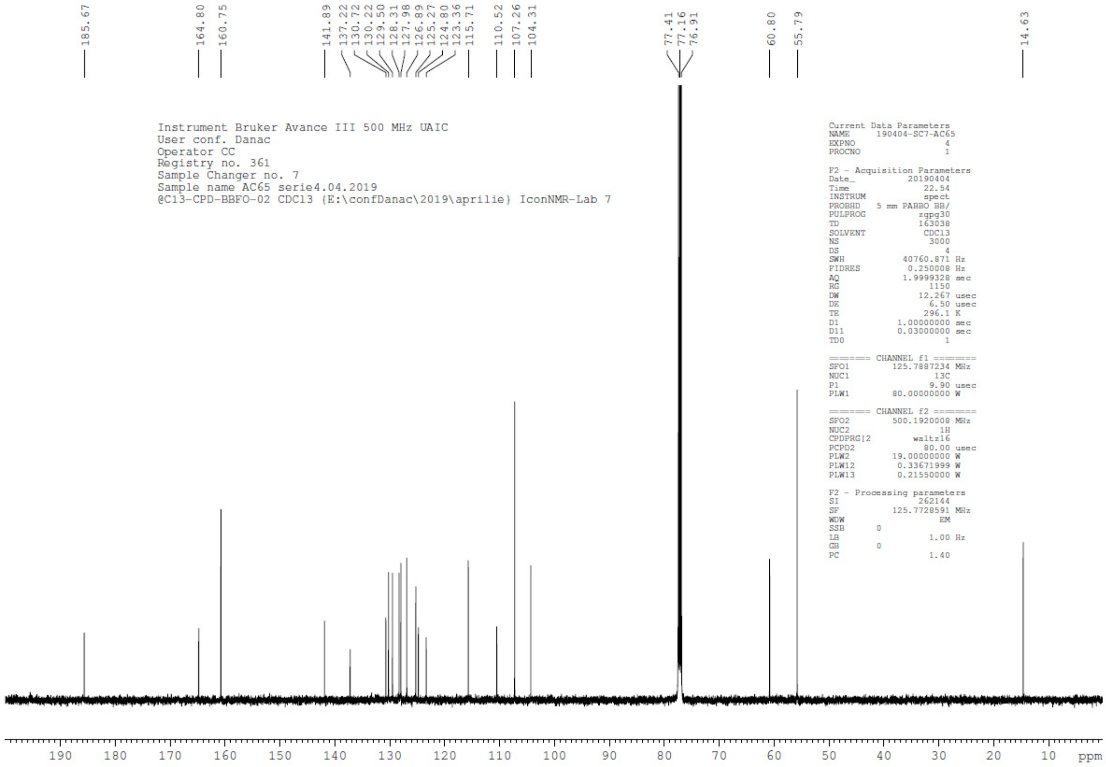

Figure S19. ^{13}C-NMR spectrum of compound **16b**

Figure S20. Results of the *in vitro* growth of cancer cell lines in the single-dose assay for compound **4b**

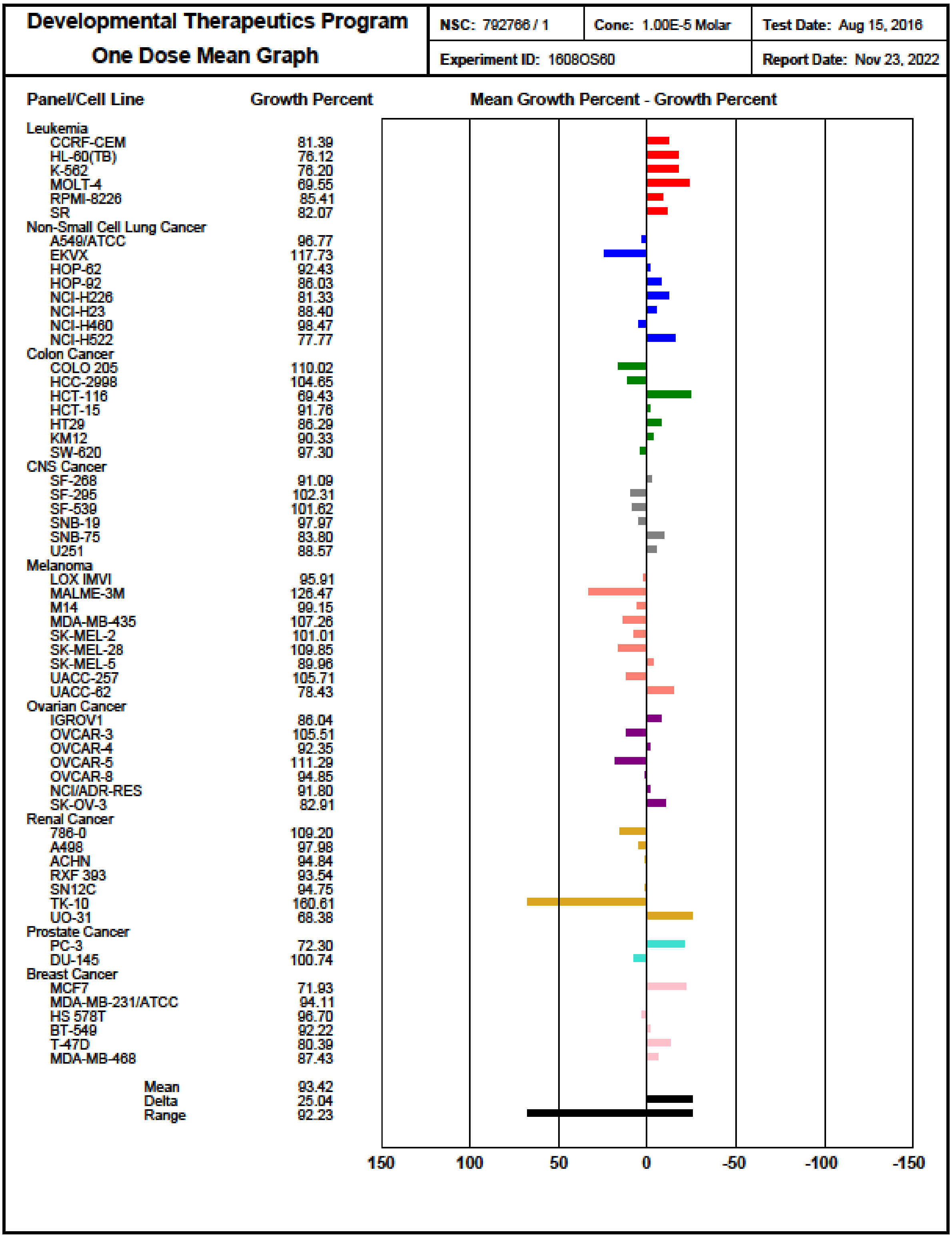

Figure S21. Results of the *in vitro* growth of cancer cell lines in the single-dose assay for compound **4c**

Developmental Therapeutics Program One Dose Mean Graph	NSC: 792778 / 1	Conc: 1.00E-5 Molar	Test Date: Aug 15, 2016
	Experiment ID: 1608OS60		Report Date: Nov 23, 2022

Panel/Cell Line	Growth Percent
**Leukemia**	
CCRF-CEM	101.23
HL-60(TB)	92.02
K-562	87.54
MOLT-4	94.24
RPMI-8226	88.25
SR	78.53
**Non-Small Cell Lung Cancer**	
A549/ATCC	99.28
EKVX	105.78
HOP-62	105.62
HOP-92	94.93
NCI-H226	100.53
NCI-H23	97.43
NCI-H460	106.20
NCI-H522	73.99
**Colon Cancer**	
COLO 205	114.54
HCC-2998	101.29
HCT-116	93.34
HCT-15	89.91
HT29	84.60
KM12	100.88
SW-620	100.42
**CNS Cancer**	
SF-268	108.63
SF-295	101.92
SF-539	99.27
SNB-19	97.95
SNB-75	104.26
U251	104.81
**Melanoma**	
LOX IMVI	77.68
MALME-3M	112.27
M14	101.96
MDA-MB-435	103.90
SK-MEL-2	96.01
SK-MEL-28	115.05
SK-MEL-5	89.41
UACC-257	102.19
UACC-62	93.99
**Ovarian Cancer**	
IGROV1	102.17
OVCAR-3	107.77
OVCAR-4	104.30
OVCAR-5	123.72
OVCAR-8	94.94
NCI/ADR-RES	96.87
SK-OV-3	64.21
**Renal Cancer**	
786-0	106.46
A498	94.59
ACHN	88.09
RXF 393	106.26
SN12C	101.68
TK-10	123.09
UO-31	71.26
**Prostate Cancer**	
PC-3	80.58
DU-145	101.95
**Breast Cancer**	
MCF7	79.60
MDA-MB-231/ATCC	91.42
HS 578T	102.40
BT-549	84.52
T-47D	78.82
MDA-MB-468	99.52
Mean	96.97
Delta	32.76
Range	59.51

Mean Growth Percent - Growth Percent

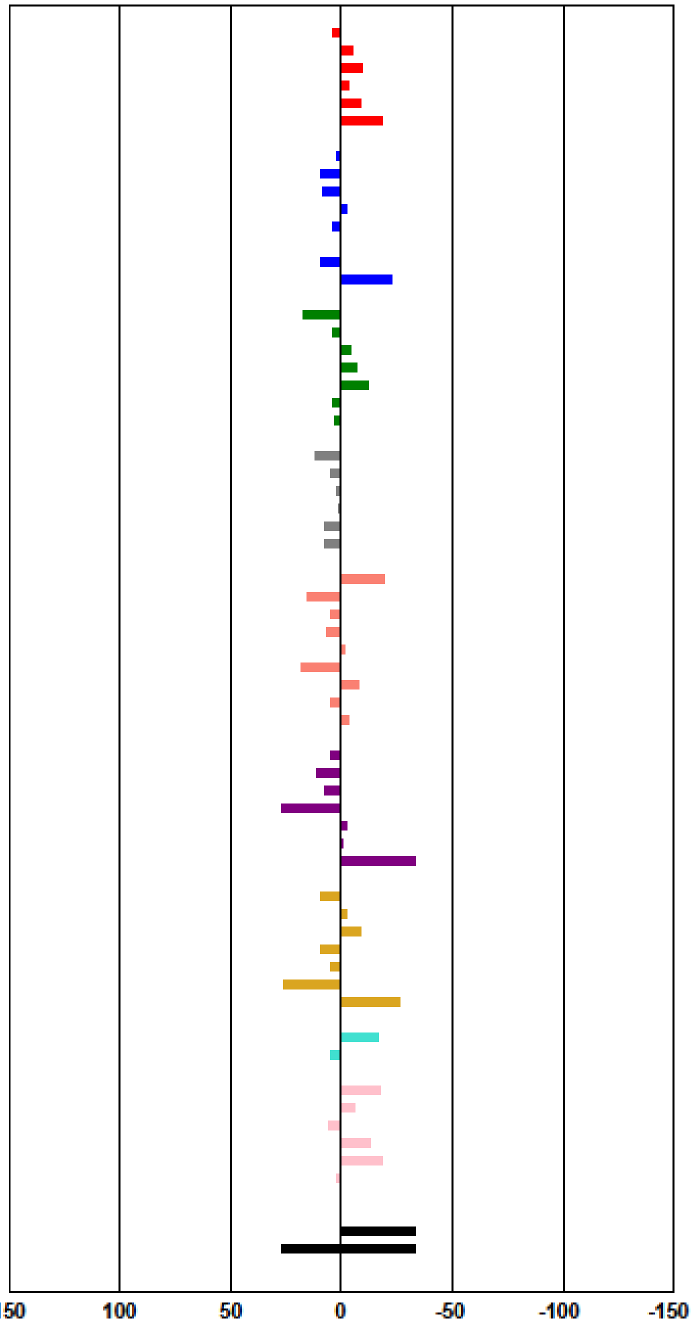

Figure S22. Results of the *in vitro* growth of cancer cell lines in the single-dose assay for compound **4d**

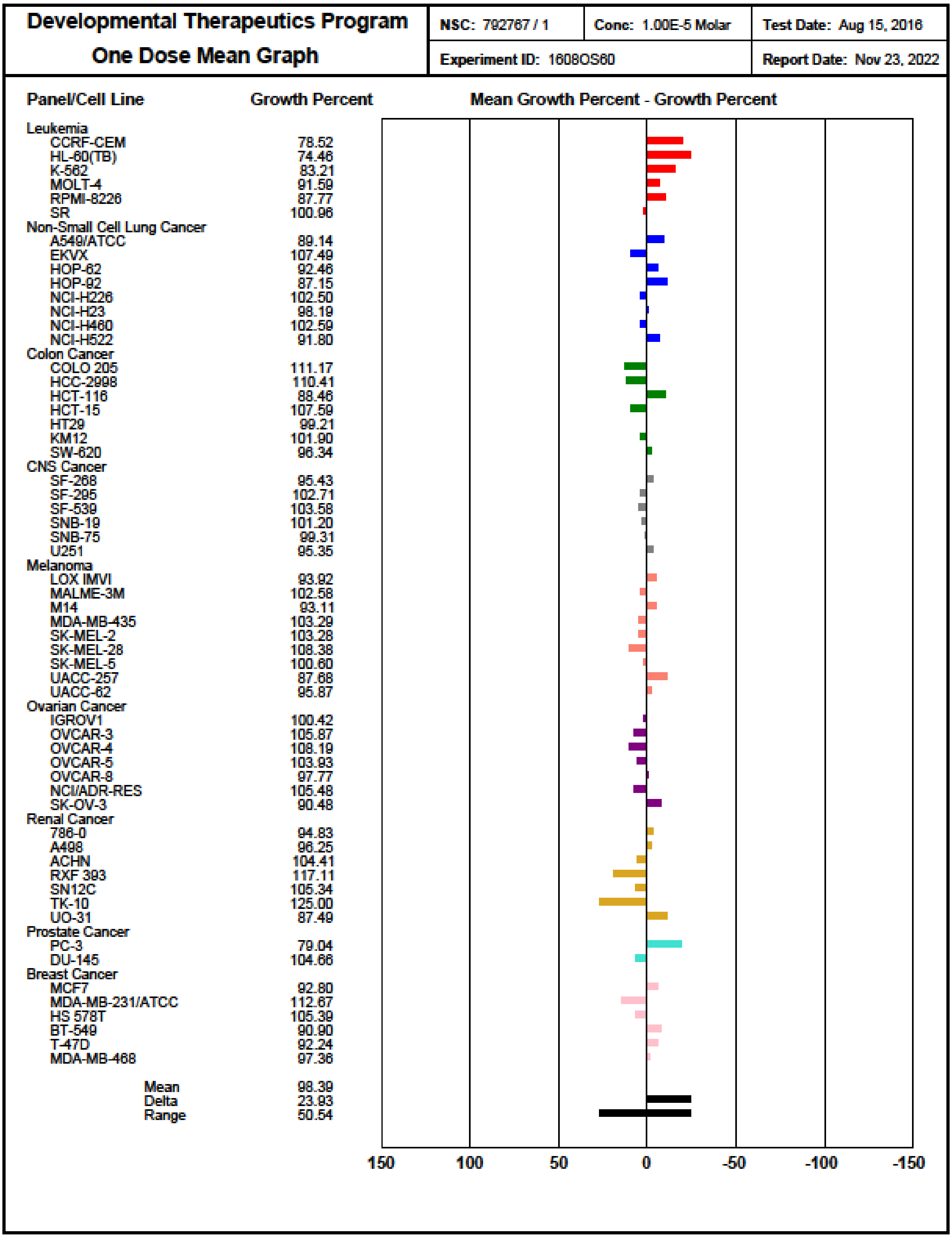

Figure S23. Results of the *in vitro* growth of cancer cell lines in the single-dose assay for compound **7b**

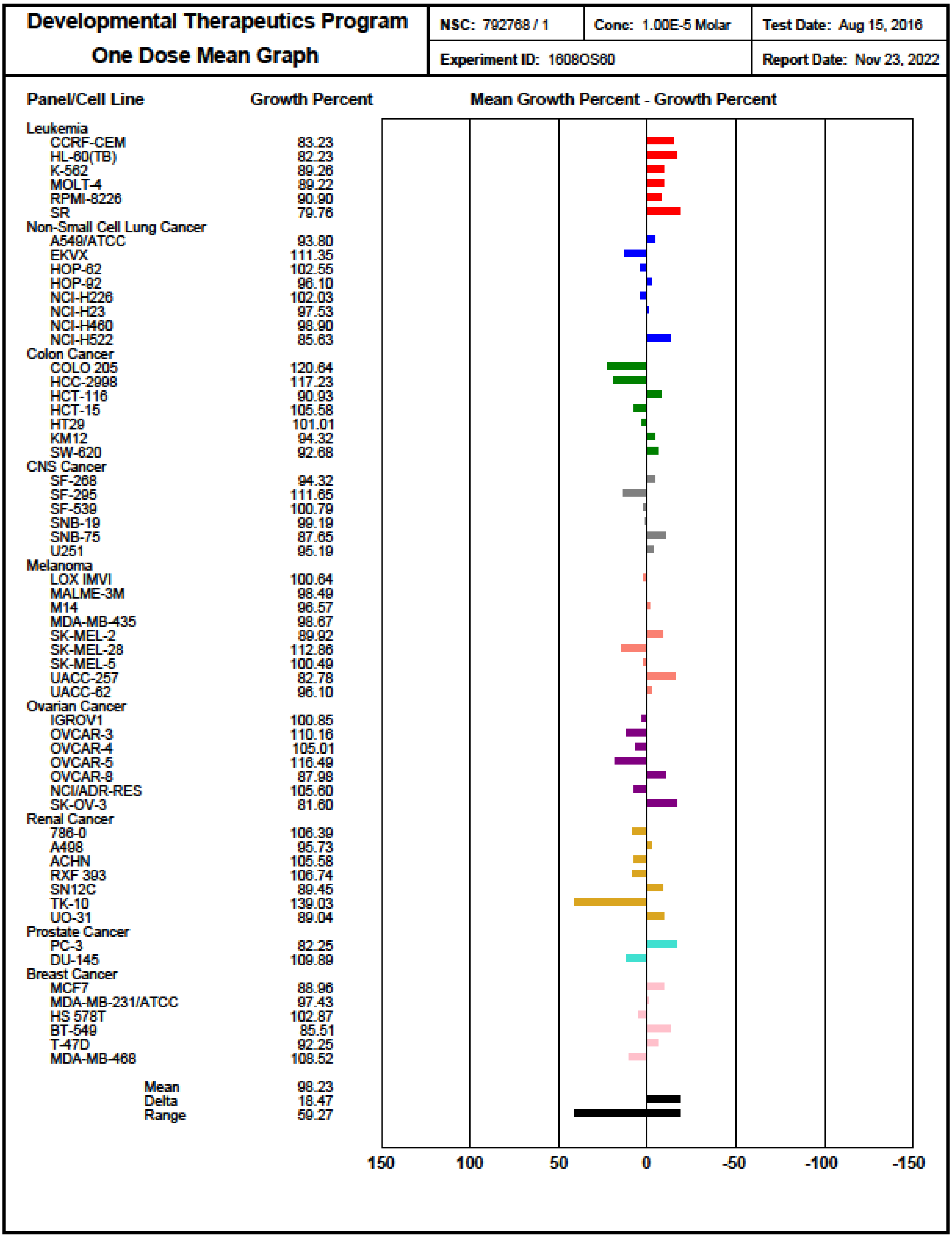

Figure S24. Results of the *in vitro* growth of cancer cell lines in the single-dose assay for compound **7c**

Developmental Therapeutics Program One Dose Mean Graph	NSC: 792779 / 1	Conc: 1.00E-5 Molar	Test Date: Aug 15, 2016
	Experiment ID: 1608OS60		Report Date: Nov 23, 2022

Panel/Cell Line	Growth Percent
**Leukemia**	
CCRF-CEM	98.78
HL-60(TB)	92.48
K-562	84.12
MOLT-4	84.30
RPMI-8226	103.97
SR	101.33
**Non-Small Cell Lung Cancer**	
A549/ATCC	85.41
EKVX	90.90
HOP-62	83.41
HOP-92	85.78
NCI-H226	93.58
NCI-H23	94.88
NCI-H460	99.36
NCI-H522	79.53
**Colon Cancer**	
COLO 205	75.11
HCC-2998	96.26
HCT-116	76.46
HCT-15	94.27
HT29	85.10
KM12	101.73
SW-620	91.09
**CNS Cancer**	
SF-268	100.00
SF-295	99.08
SF-539	93.79
SNB-19	102.98
SNB-75	83.44
U251	94.80
**Melanoma**	
LOX IMVI	97.13
MALME-3M	84.80
M14	90.15
MDA-MB-435	94.88
SK-MEL-2	100.08
SK-MEL-28	96.95
SK-MEL-5	92.95
UACC-257	90.73
UACC-62	91.44
**Ovarian Cancer**	
IGROV1	94.23
OVCAR-3	109.61
OVCAR-4	92.92
OVCAR-5	100.54
OVCAR-8	105.41
NCI/ADR-RES	100.41
SK-OV-3	76.78
**Renal Cancer**	
786-0	93.80
A498	95.42
ACHN	95.97
RXF 393	96.78
SN12C	91.11
TK-10	104.94
UO-31	79.08
**Prostate Cancer**	
PC-3	76.89
DU-145	102.56
**Breast Cancer**	
MCF7	86.87
MDA-MB-231/ATCC	101.51
HS 578T	96.98
BT-549	78.44
T-47D	76.66
MDA-MB-468	100.40
Mean	92.56
Delta	17.45
Range	34.50

Mean Growth Percent - Growth Percent

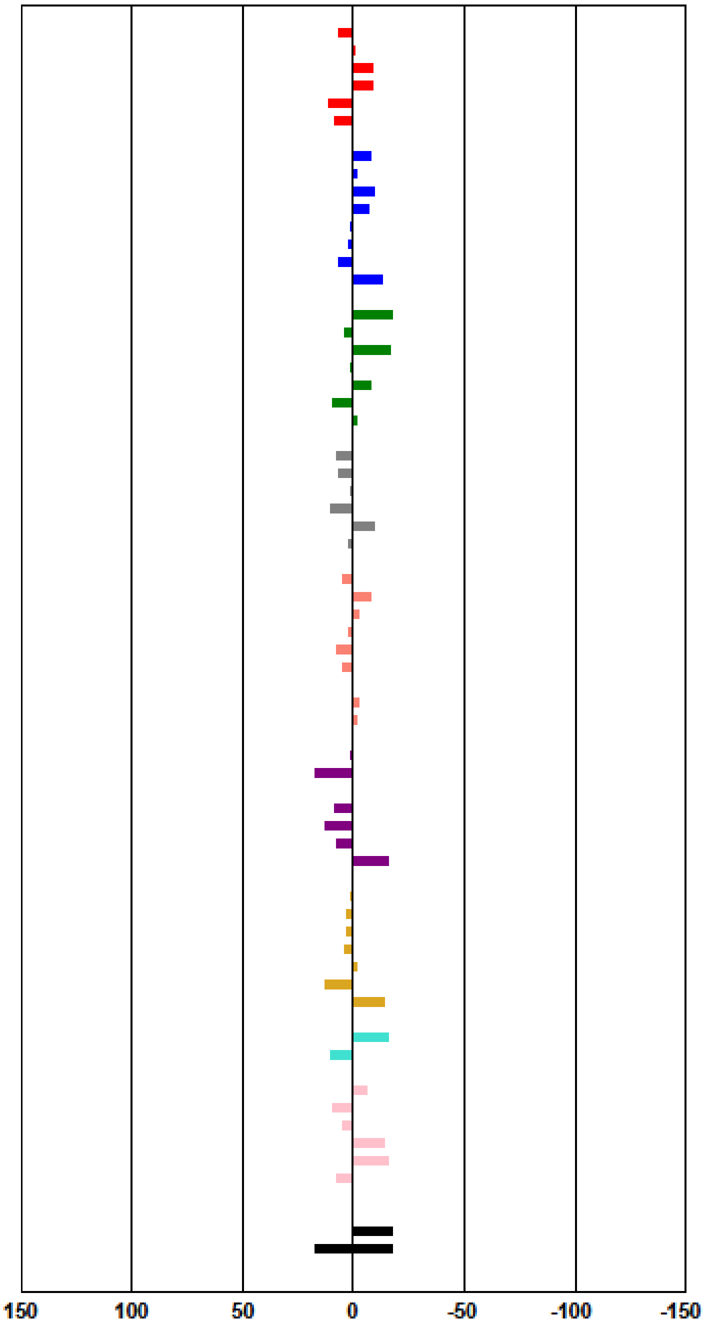

Figure S25. Results of the *in vitro* growth of cancer cell lines in the single-dose assay for compound **9a**

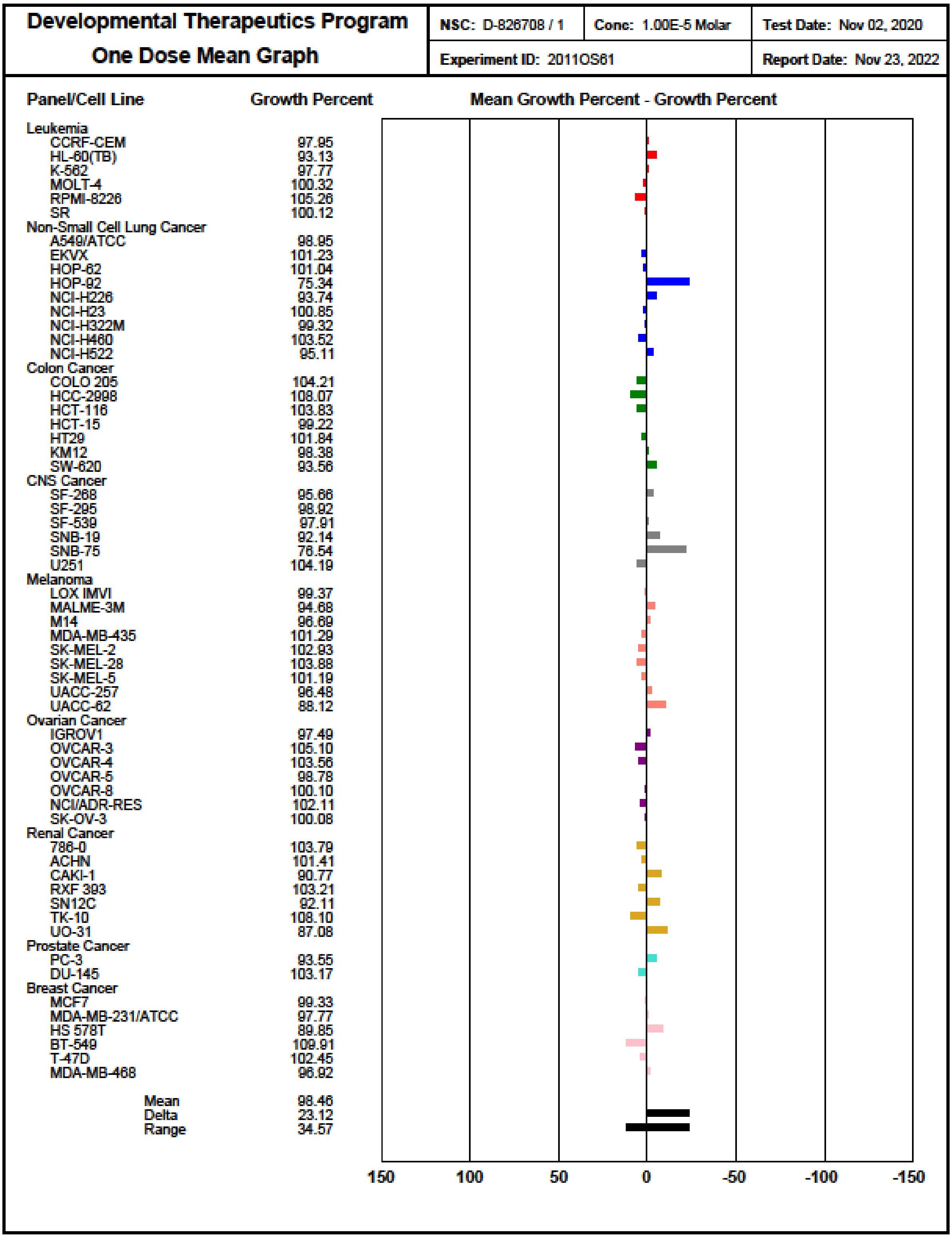

Figure S26. Results of the *in vitro* growth of cancer cell lines in the single-dose assay for compound **9d**

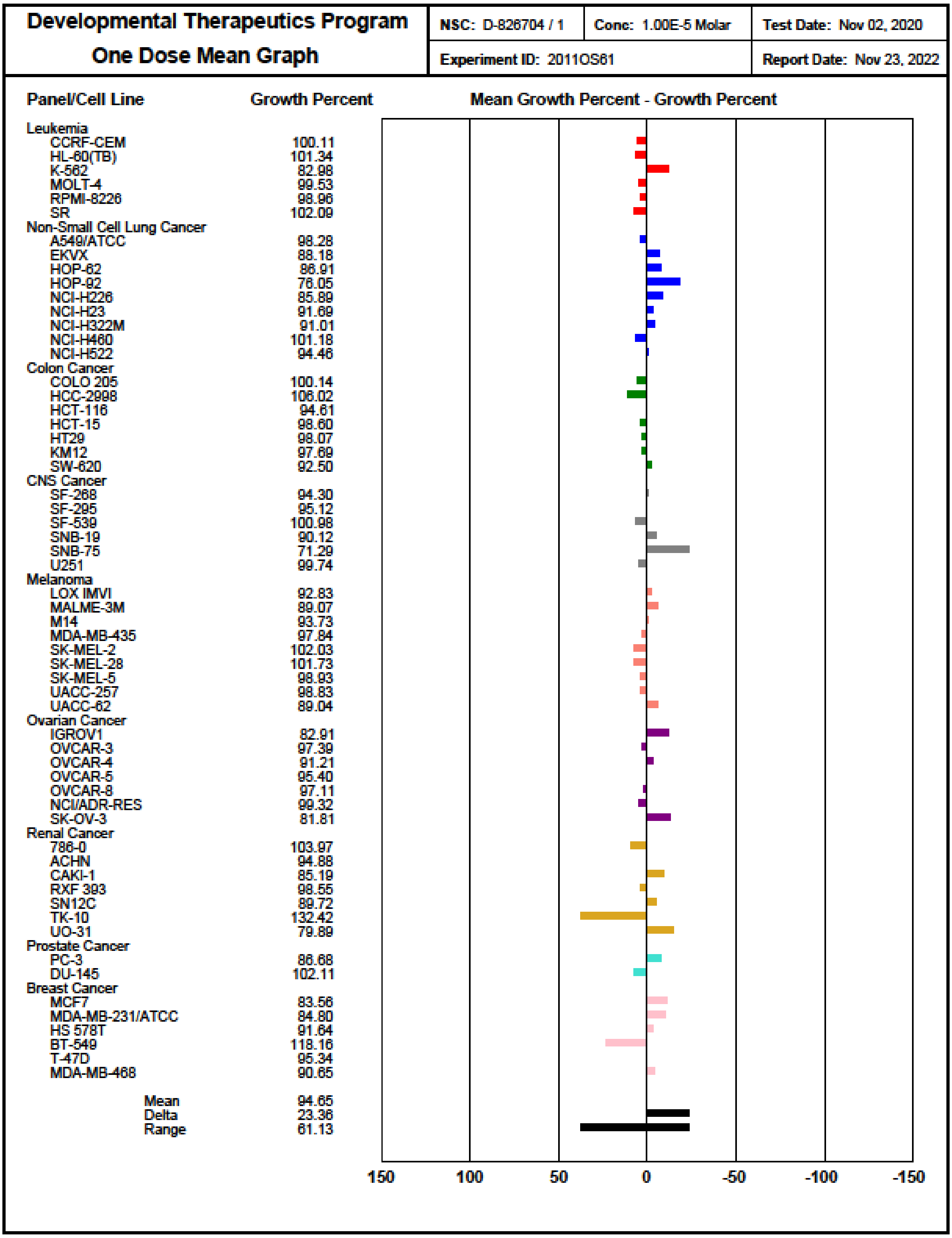

Figure S27. Results of the *in vitro* growth of cancer cell lines in the single-dose assay for compound **10a**

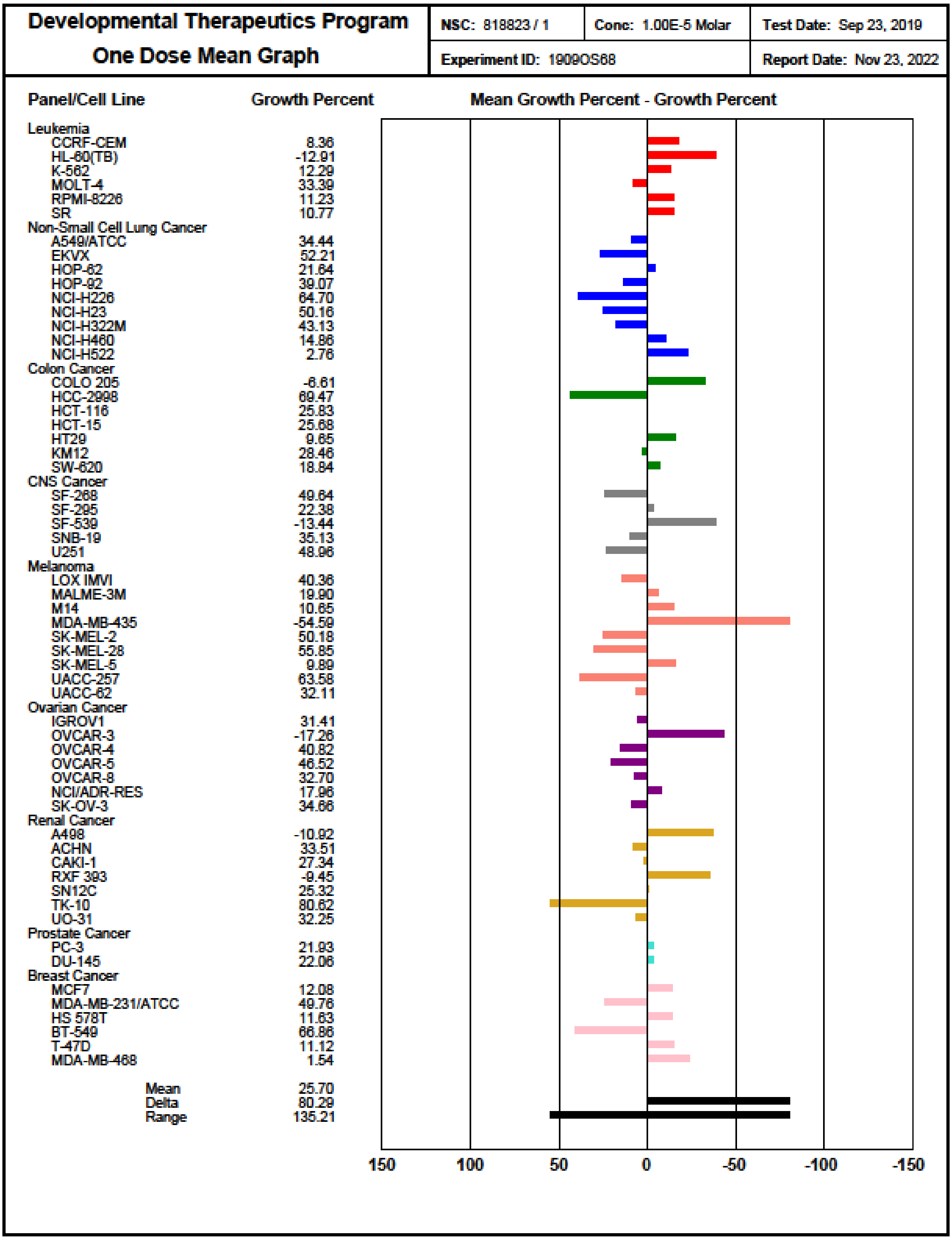

Figure S28. Results of the *in vitro* growth of cancer cell lines in the single-dose assay for **10b**

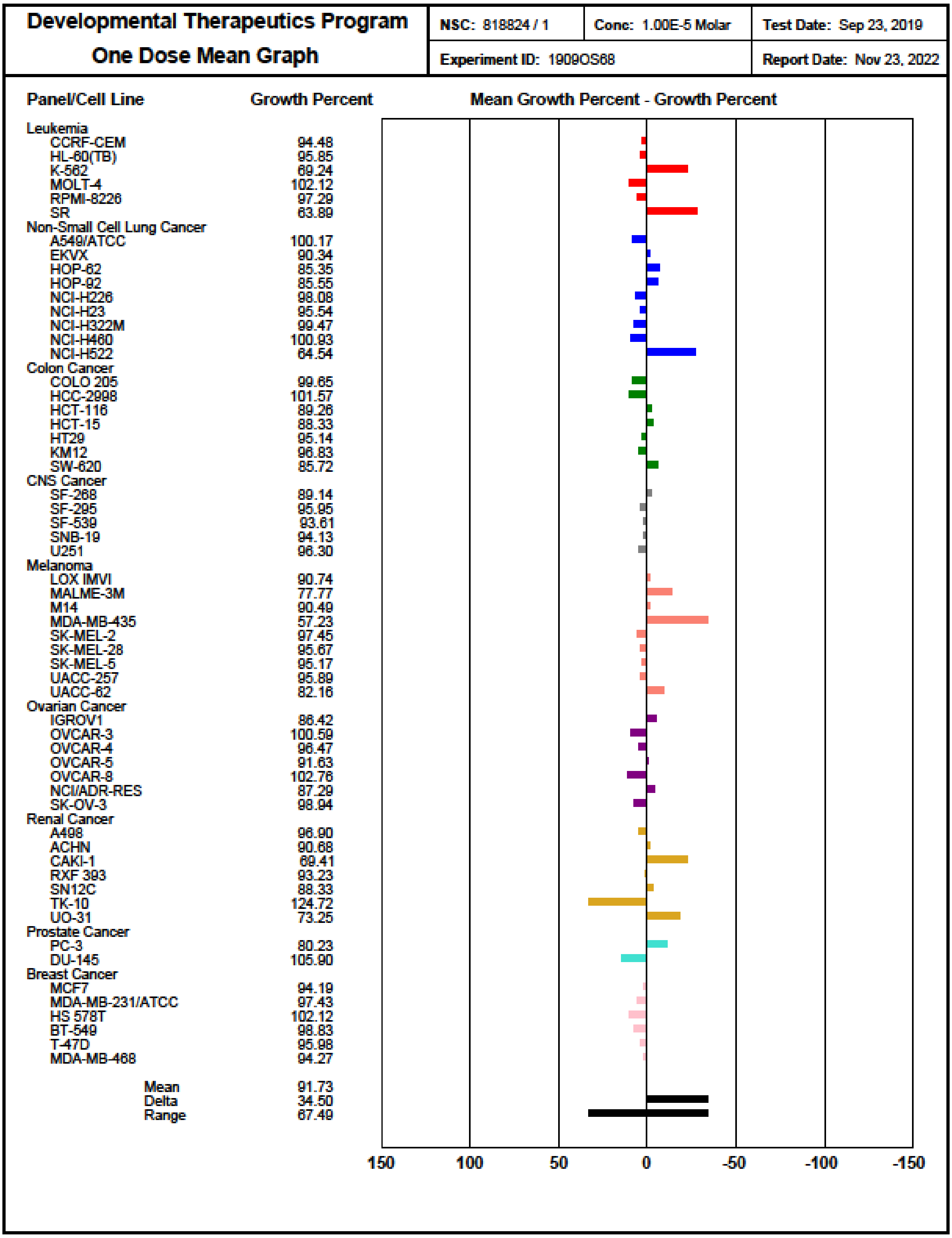

Figure S29. Results of the *in vitro* growth of cancer cell lines in the single-dose assay for **10c**

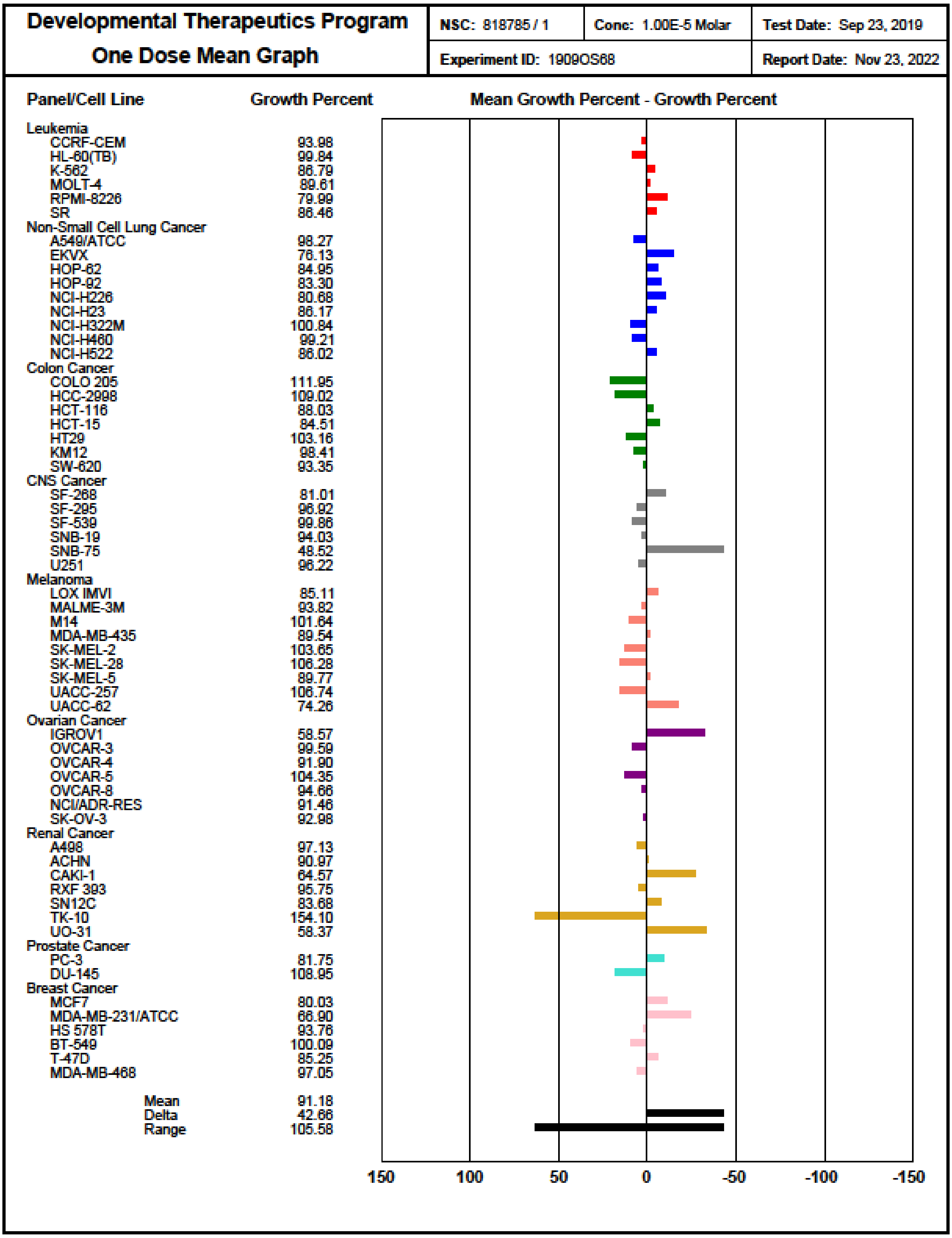

Figure S30. Results of the *in vitro* growth of cancer cell lines in the single-dose assay for **10d**

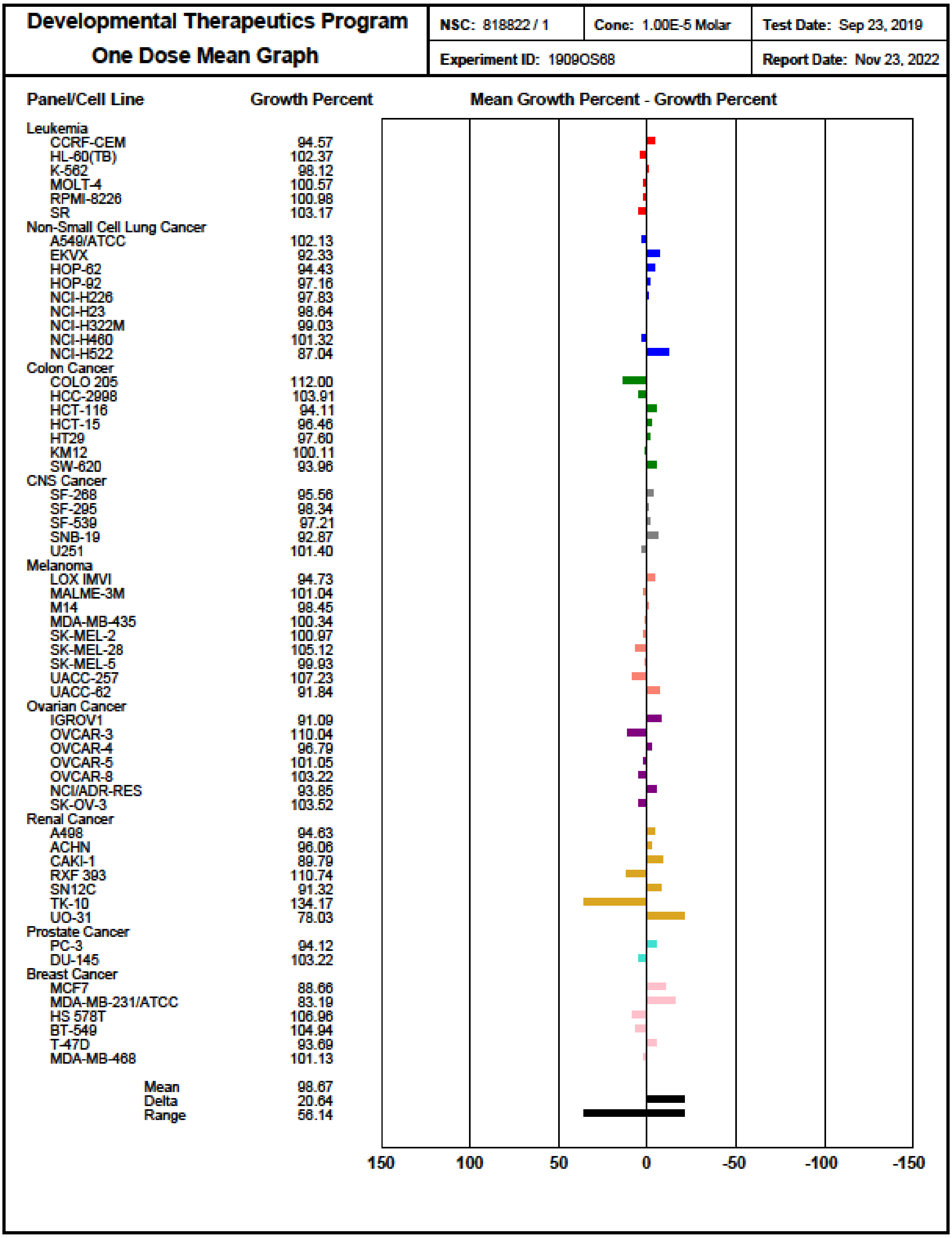

Figure S31. Results of the *in vitro* growth of cancer cell lines in the single-dose assay for **12a**

Developmental Therapeutics Program
One Dose Mean Graph

NSC: D-826104 / 1	Conc: 1.00E-5 Molar	Test Date: Sep 28, 2020
Experiment ID: 2009OS51		Report Date: Nov 23, 2022

Panel/Cell Line	Growth Percent
**Leukemia**	
CCRF-CEM	94.66
HL-60(TB)	91.85
K-562	67.15
MOLT-4	99.11
SR	100.94
**Non-Small Cell Lung Cancer**	
A549/ATCC	89.74
EKVX	90.92
HOP-62	95.15
HOP-92	72.77
NCI-H226	94.05
NCI-H23	91.01
NCI-H322M	97.52
NCI-H460	90.60
NCI-H522	84.44
**Colon Cancer**	
COLO 205	99.65
HCC-2998	91.57
HCT-116	39.07
HCT-15	102.48
HT29	82.64
KM12	92.86
SW-620	98.49
**CNS Cancer**	
SF-268	90.36
SF-295	91.90
SF-539	101.11
SNB-19	92.32
SNB-75	90.27
U251	87.83
**Melanoma**	
LOX IMVI	98.09
MALME-3M	91.69
M14	99.12
SK-MEL-2	102.10
SK-MEL-28	101.29
SK-MEL-5	96.33
UACC-257	94.98
UACC-62	91.20
**Ovarian Cancer**	
IGROV1	98.48
OVCAR-3	87.53
OVCAR-4	78.25
OVCAR-5	101.47
OVCAR-8	91.29
NCI/ADR-RES	104.09
SK-OV-3	102.75
**Renal Cancer**	
786-0	96.25
A498	106.34
ACHN	101.31
CAKI-1	96.86
RXF 393	104.16
SN12C	90.65
TK-10	101.15
UO-31	90.38
**Prostate Cancer**	
PC-3	87.87
DU-145	99.73
**Breast Cancer**	
MCF7	77.59
MDA-MB-231/ATCC	89.69
HS 578T	99.25
BT-549	102.13
T-47D	76.50
MDA-MB-468	58.01
Mean	92.02
Delta	52.95
Range	67.27

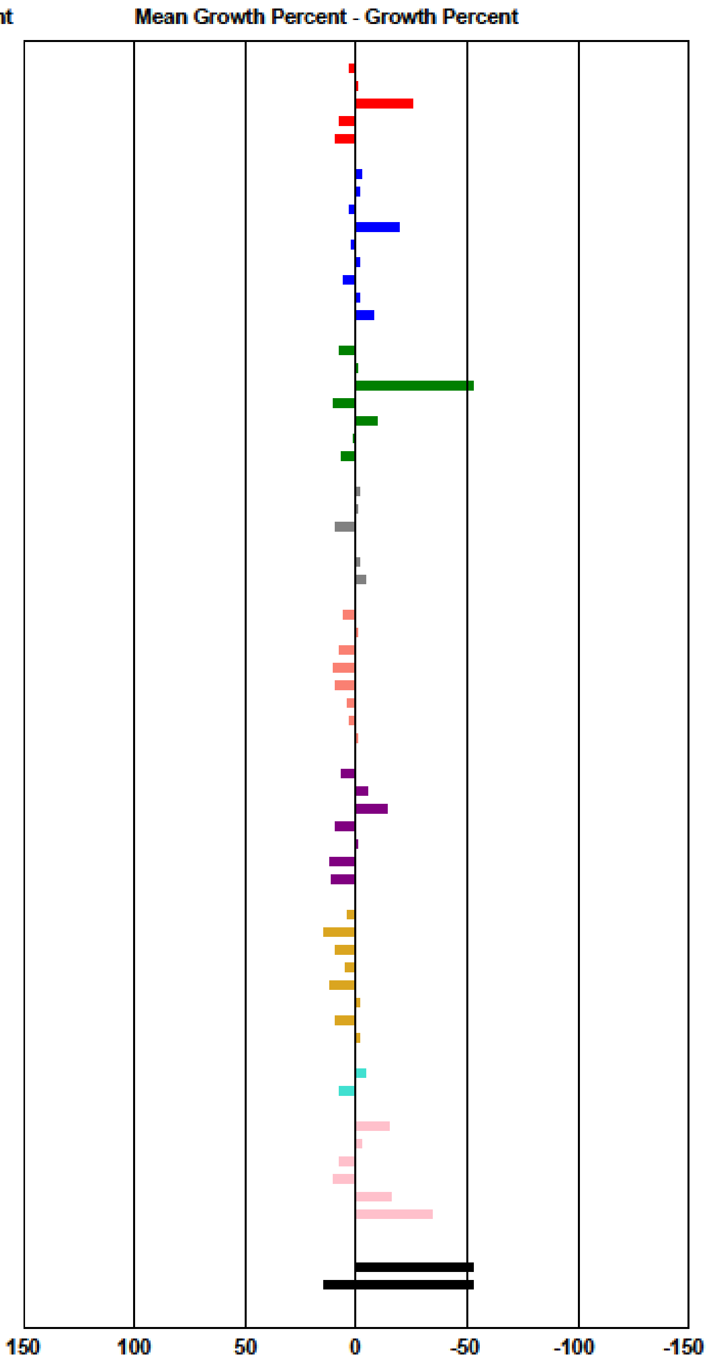

Figure S32. Results of the *in vitro* growth of cancer cell lines in the single-dose assay for **12b**

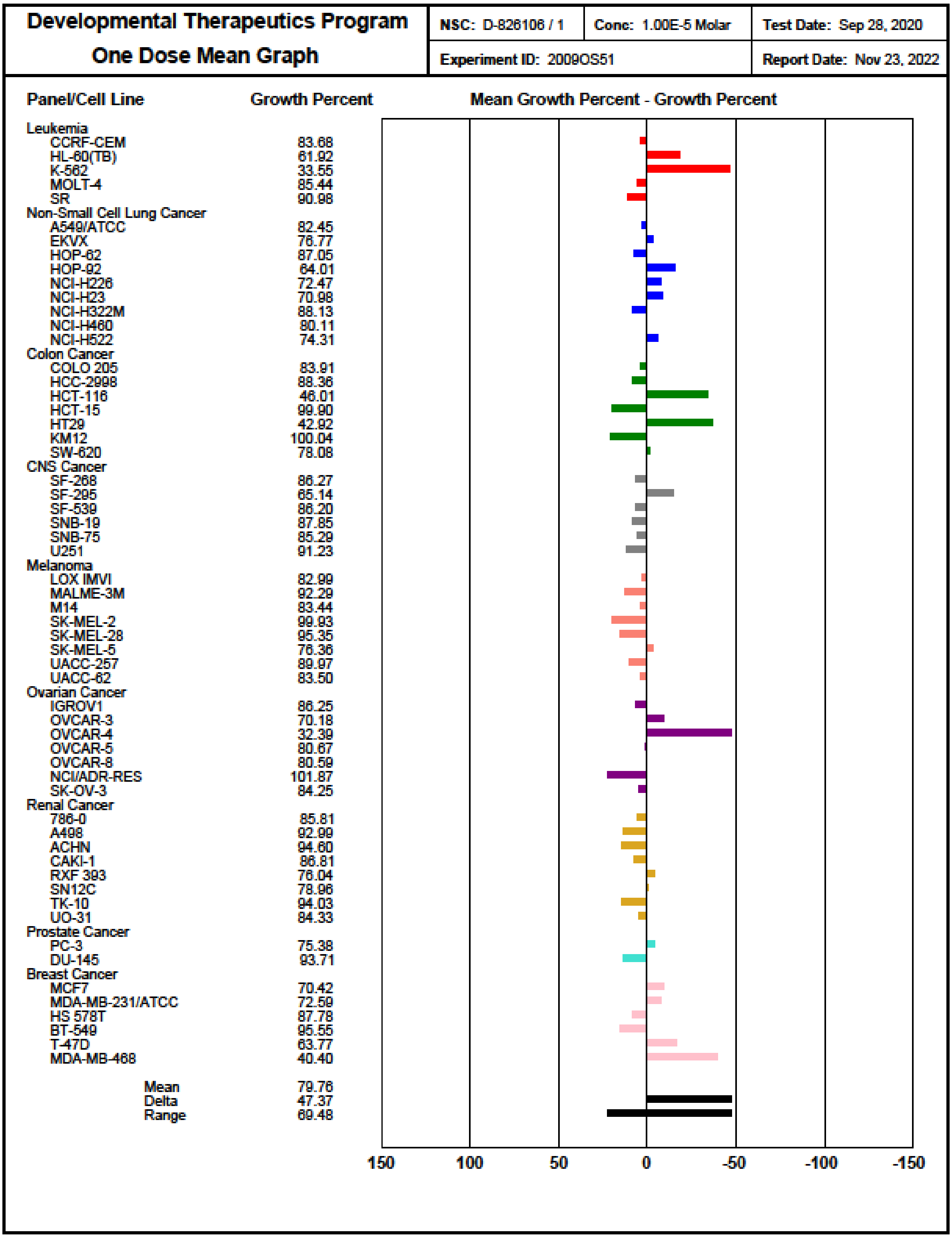

Figure S33. Results of the *in vitro* growth of cancer cell lines in the single-dose assay for **12c**

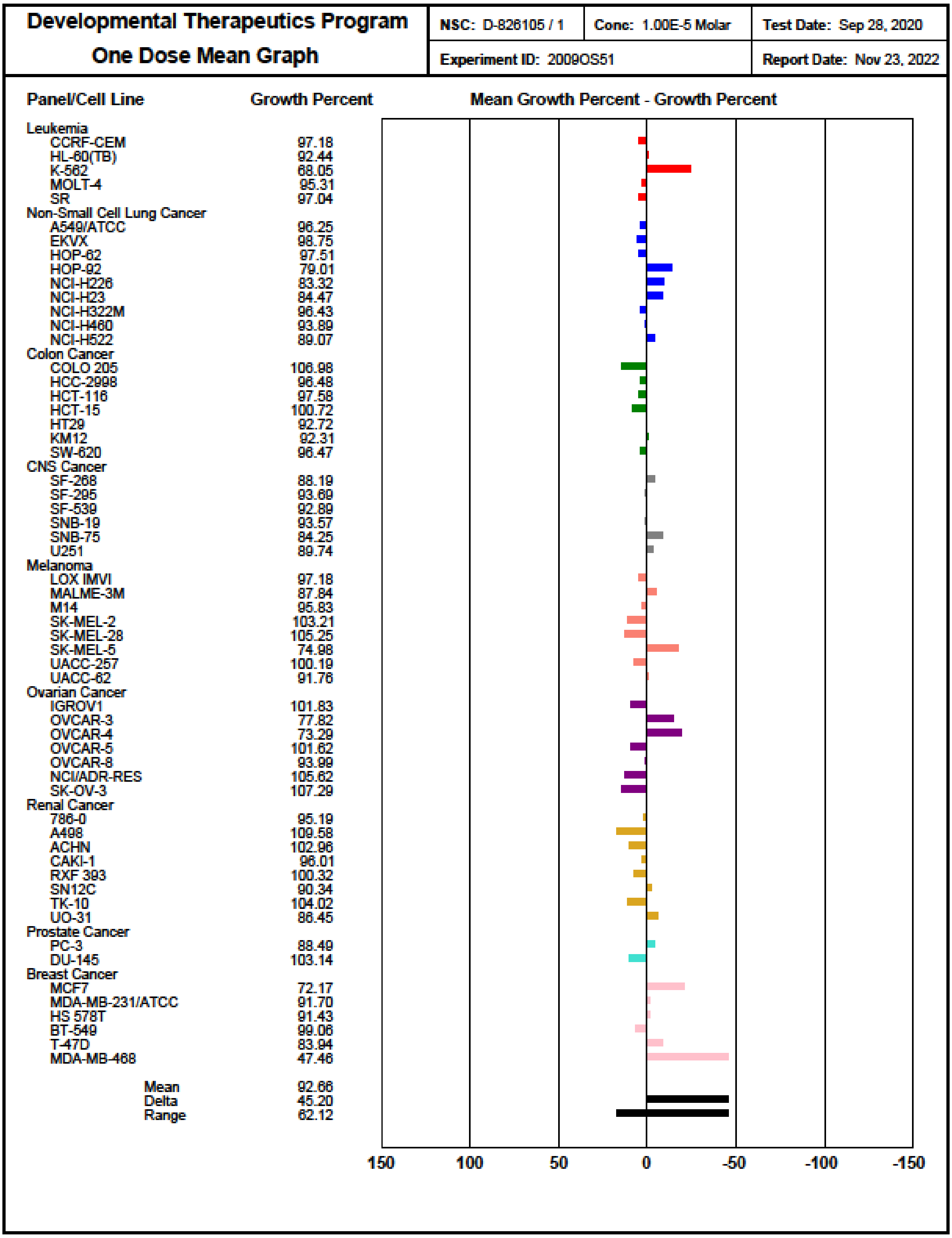

Figure S34. Results of the *in vitro* growth of cancer cell lines in the single-dose assay for **12d**

Developmental Therapeutics Program One Dose Mean Graph	NSC: D-826103 / 1	Conc: 1.00E-5 Molar	Test Date: Sep 28, 2020
	Experiment ID: 2009OS51		Report Date: Nov 23, 2022

Panel/Cell Line	Growth Percent
Leukemia	
CCRF-CEM	82.53
HL-60(TB)	62.45
K-562	34.55
MOLT-4	75.83
SR	85.04
Non-Small Cell Lung Cancer	
A549/ATCC	84.11
EKVX	82.40
HOP-62	75.91
HOP-92	50.88
NCI-H226	71.79
NCI-H23	76.37
NCI-H322M	91.55
NCI-H460	82.96
NCI-H522	80.82
Colon Cancer	
COLO 205	81.39
HCC-2998	82.95
HCT-116	51.38
HCT-15	102.02
HT29	49.76
KM12	88.06
SW-620	76.51
CNS Cancer	
SF-268	81.89
SF-295	67.61
SF-539	84.13
SNB-19	89.30
SNB-75	69.94
U251	95.16
Melanoma	
LOX IMVI	82.14
MALME-3M	87.32
M14	84.03
SK-MEL-2	103.13
SK-MEL-28	98.37
SK-MEL-5	80.23
UACC-257	91.56
UACC-62	86.63
Ovarian Cancer	
IGROV1	81.11
OVCAR-3	68.88
OVCAR-4	66.78
OVCAR-5	82.71
OVCAR-8	75.65
NCI/ADR-RES	101.49
SK-OV-3	86.72
Renal Cancer	
786-0	83.75
A498	80.15
ACHN	89.01
CAKI-1	80.25
RXF 393	67.67
SN12C	84.85
TK-10	113.95
UO-31	59.04
Prostate Cancer	
PC-3	68.85
DU-145	91.65
Breast Cancer	
MCF7	69.16
MDA-MB-231/ATCC	70.64
HS 578T	89.03
BT-549	88.35
T-47D	76.76
MDA-MB-468	54.99
Mean	79.35
Delta	44.80
Range	79.40

Mean Growth Percent - Growth Percent

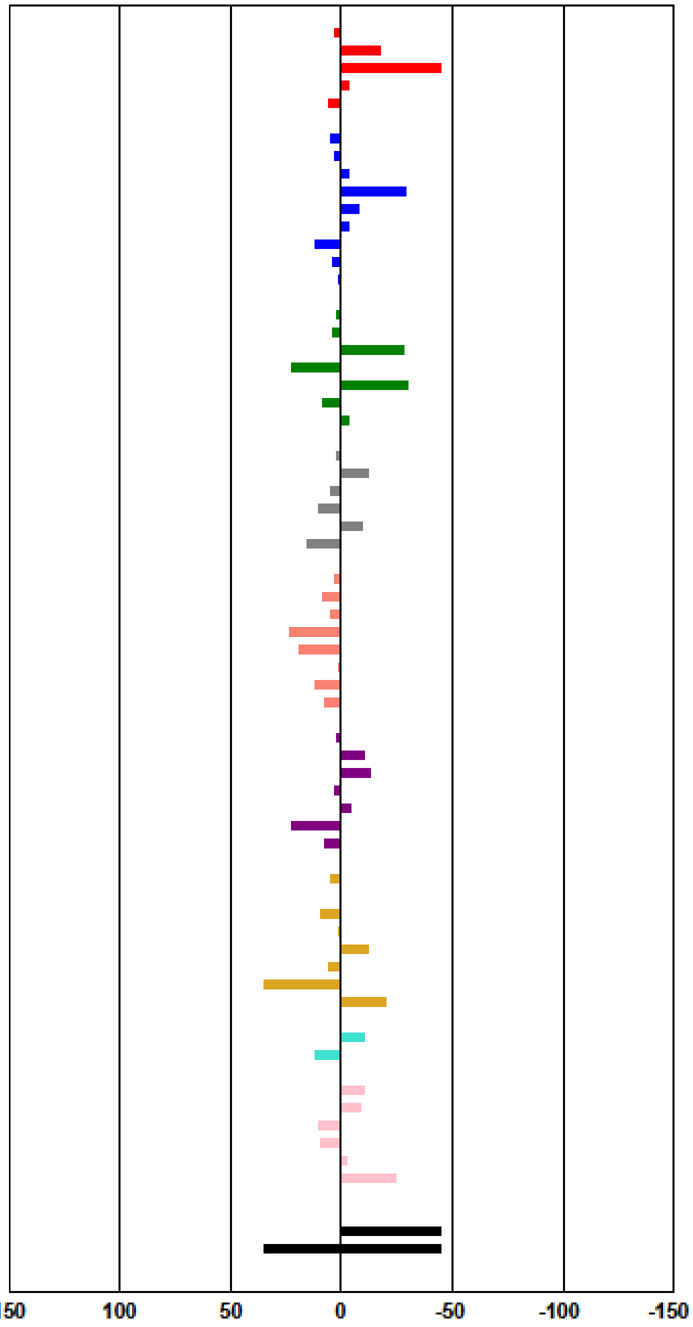

Figure S35. Results of the *in vitro* growth of cancer cell lines in the single-dose assay for **13a**

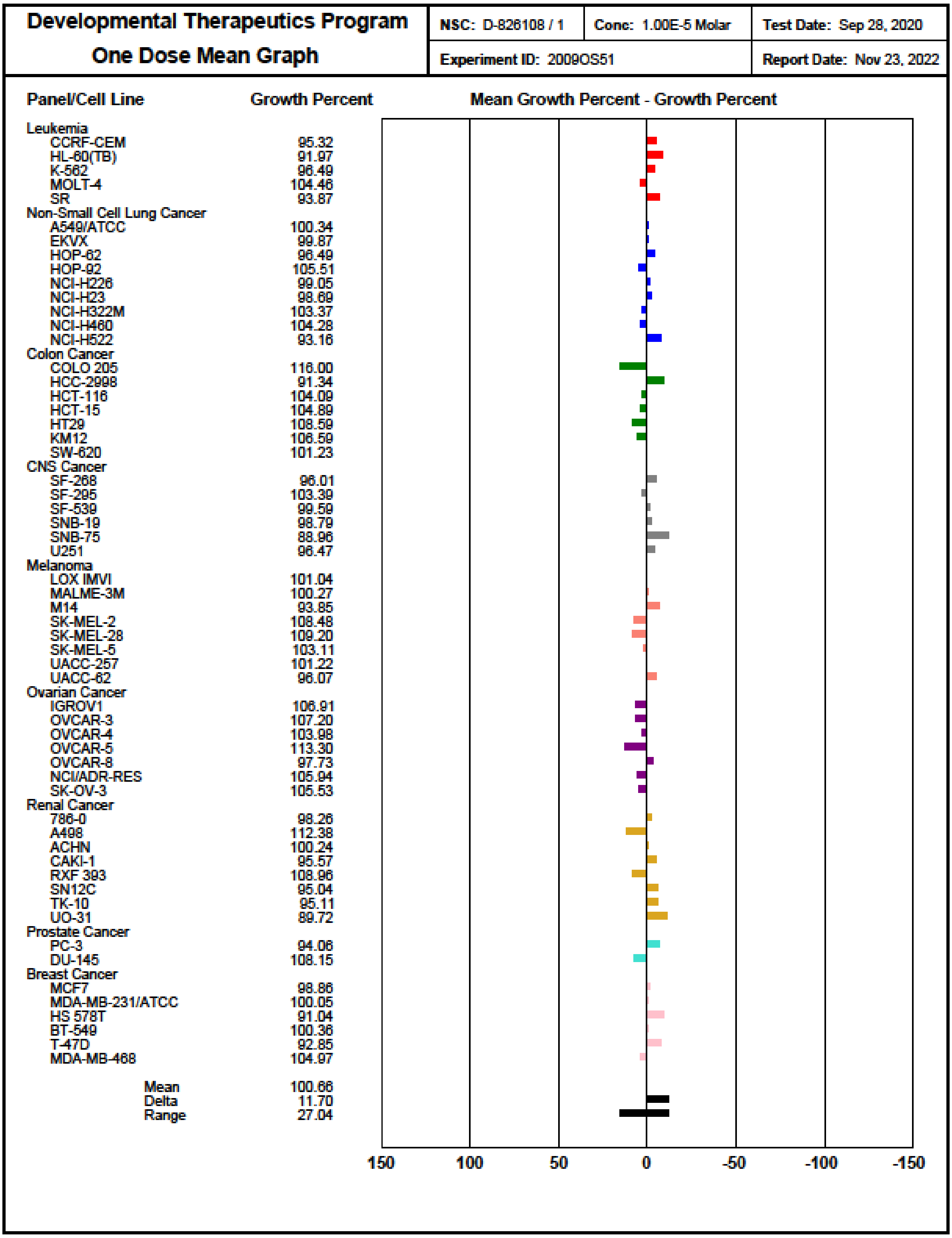

Figure S36. Results of the *in vitro* growth of cancer cell lines in the single-dose assay for **13b**

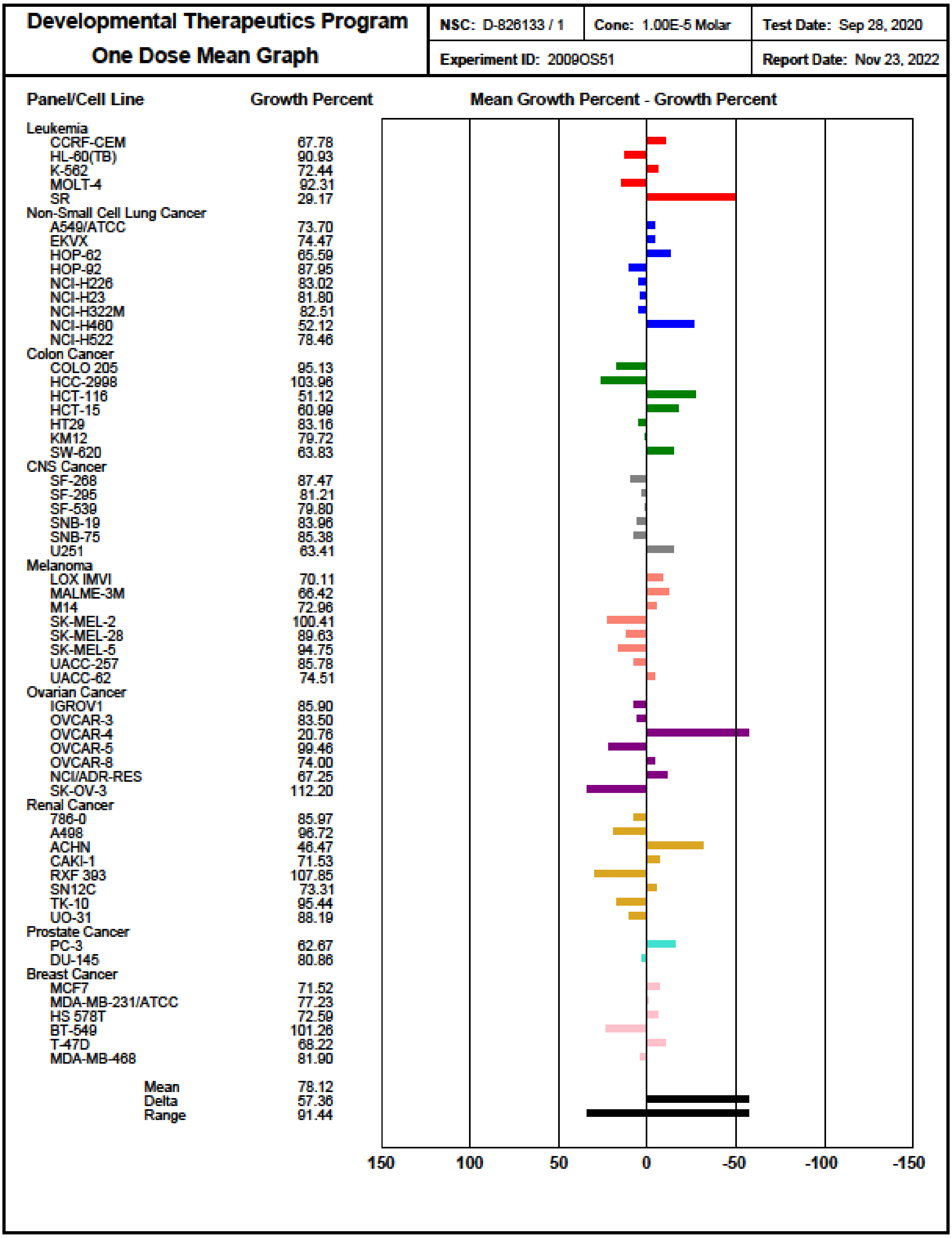

Figure S37. Results of the *in vitro* growth of cancer cell lines in the single-dose assay for **13c**

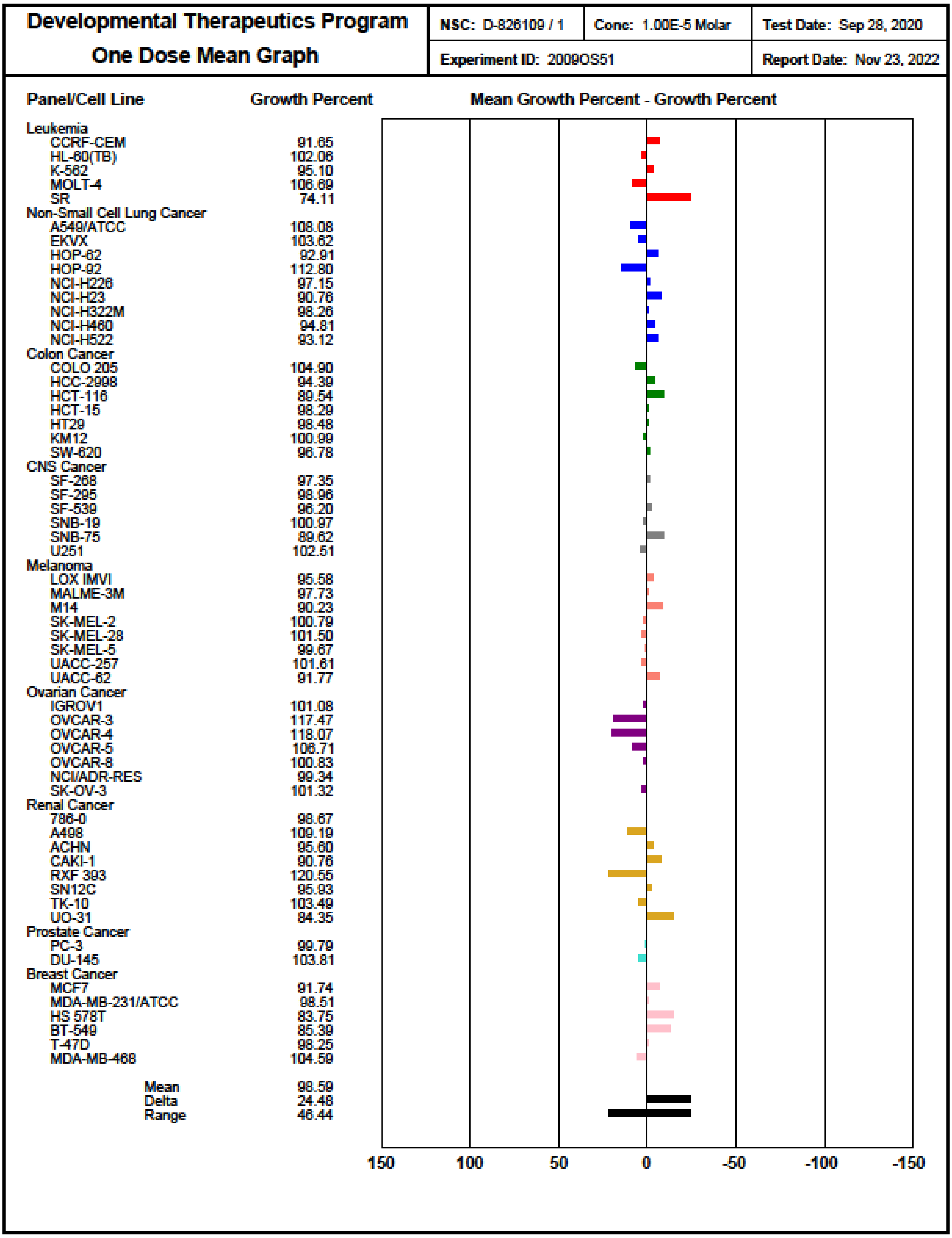

Figure S38. Results of the *in vitro* growth of cancer cell lines in the single-dose assay for **13d**

Developmental Therapeutics Program One Dose Mean Graph	NSC: D-826107 / 1	Conc: 1.00E-5 Molar	Test Date: Sep 28, 2020
	Experiment ID: 2009OS51		Report Date: Nov 23, 2022

Panel/Cell Line	Growth Percent
**Leukemia**	
CCRF-CEM	97.25
HL-60(TB)	94.55
K-562	97.09
MOLT-4	104.40
SR	94.98
**Non-Small Cell Lung Cancer**	
A549/ATCC	99.17
EKVX	99.44
HOP-62	82.04
HOP-92	98.29
NCI-H226	92.84
NCI-H23	88.54
NCI-H322M	100.85
NCI-H460	102.31
NCI-H522	90.91
**Colon Cancer**	
COLO 205	110.28
HCC-2998	101.63
HCT-116	94.06
HCT-15	96.73
HT29	99.89
KM12	99.30
SW-620	96.75
**CNS Cancer**	
SF-268	92.23
SF-295	85.07
SF-539	86.78
SNB-19	86.85
SNB-75	76.75
U251	99.72
**Melanoma**	
LOX IMVI	95.99
MALME-3M	95.15
M14	97.28
SK-MEL-2	99.95
SK-MEL-28	104.16
SK-MEL-5	101.21
UACC-257	103.97
UACC-62	90.93
**Ovarian Cancer**	
IGROV1	99.04
OVCAR-3	112.60
OVCAR-4	100.08
OVCAR-5	108.42
OVCAR-8	101.67
NCI/ADR-RES	98.91
SK-OV-3	95.55
**Renal Cancer**	
786-0	98.47
A498	115.34
ACHN	94.32
CAKI-1	103.43
RXF 393	97.89
SN12C	98.02
TK-10	109.54
UO-31	85.06
**Prostate Cancer**	
PC-3	93.05
DU-145	102.59
**Breast Cancer**	
MCF7	92.98
MDA-MB-231/ATCC	93.04
HS 578T	82.09
BT-549	97.59
T-47D	94.92
MDA-MB-468	99.45
Mean	97.09
Delta	20.34
Range	38.59

Mean Growth Percent - Growth Percent

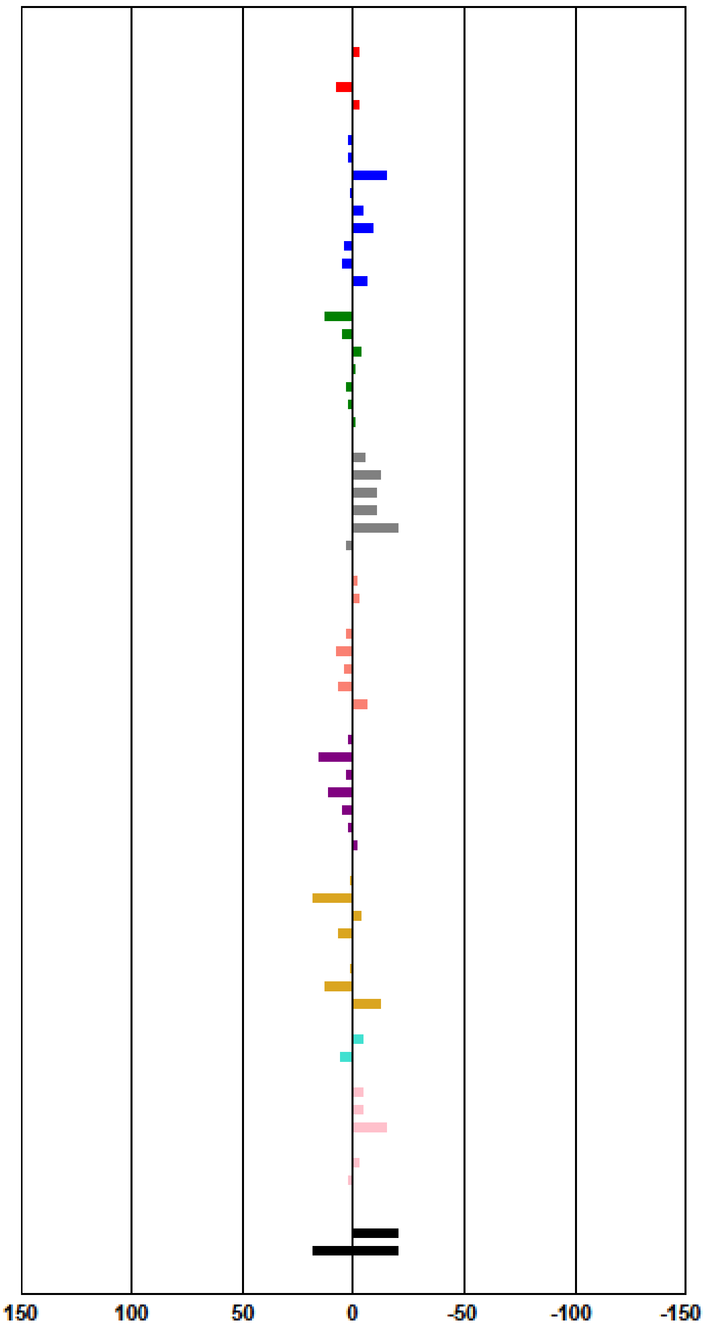

Figure S39. Results of the *in vitro* growth of cancer cell lines in the single-dose assay for **15a**

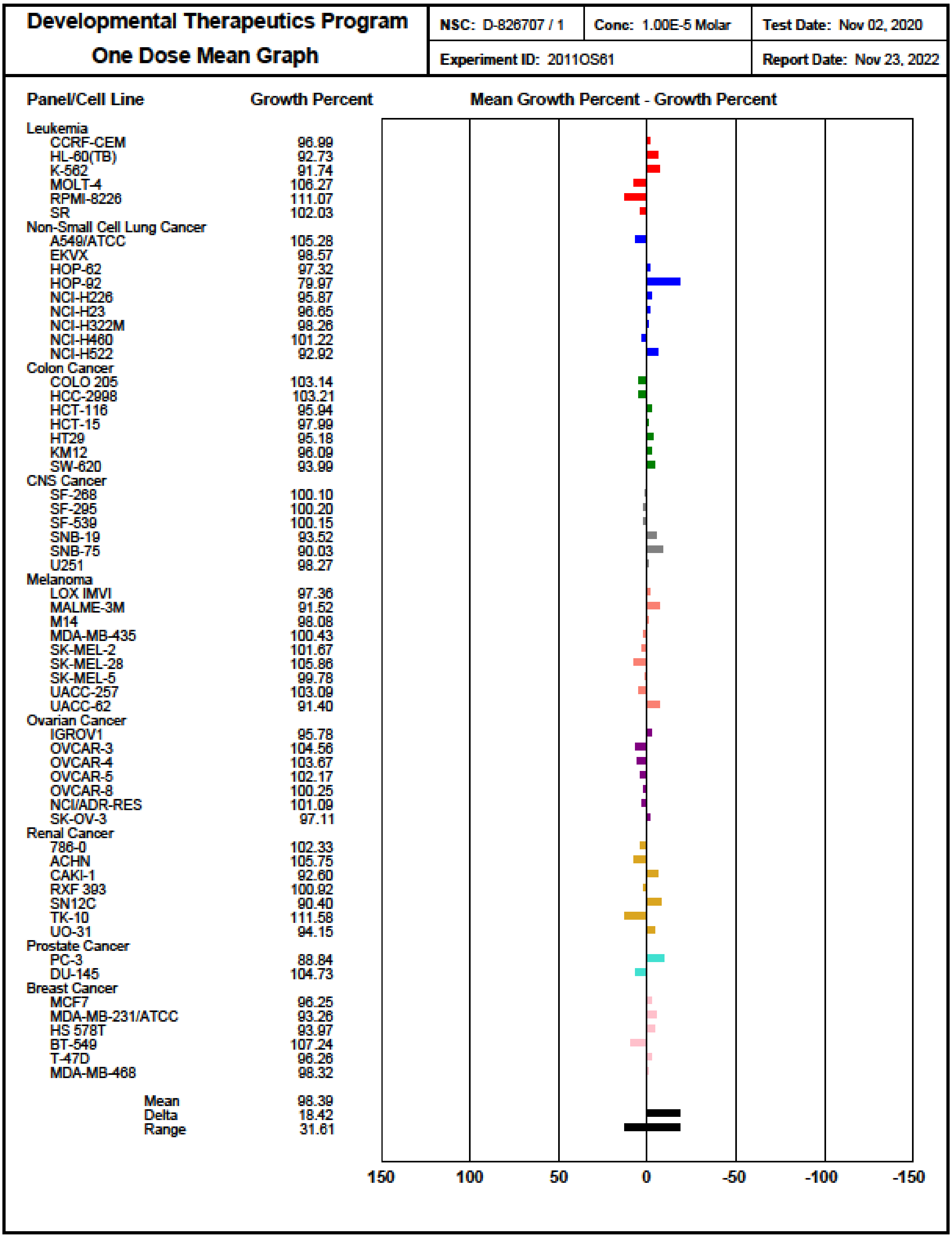

Figure S40. Results of the *in vitro* growth of cancer cell lines in the single-dose assay for **15d**

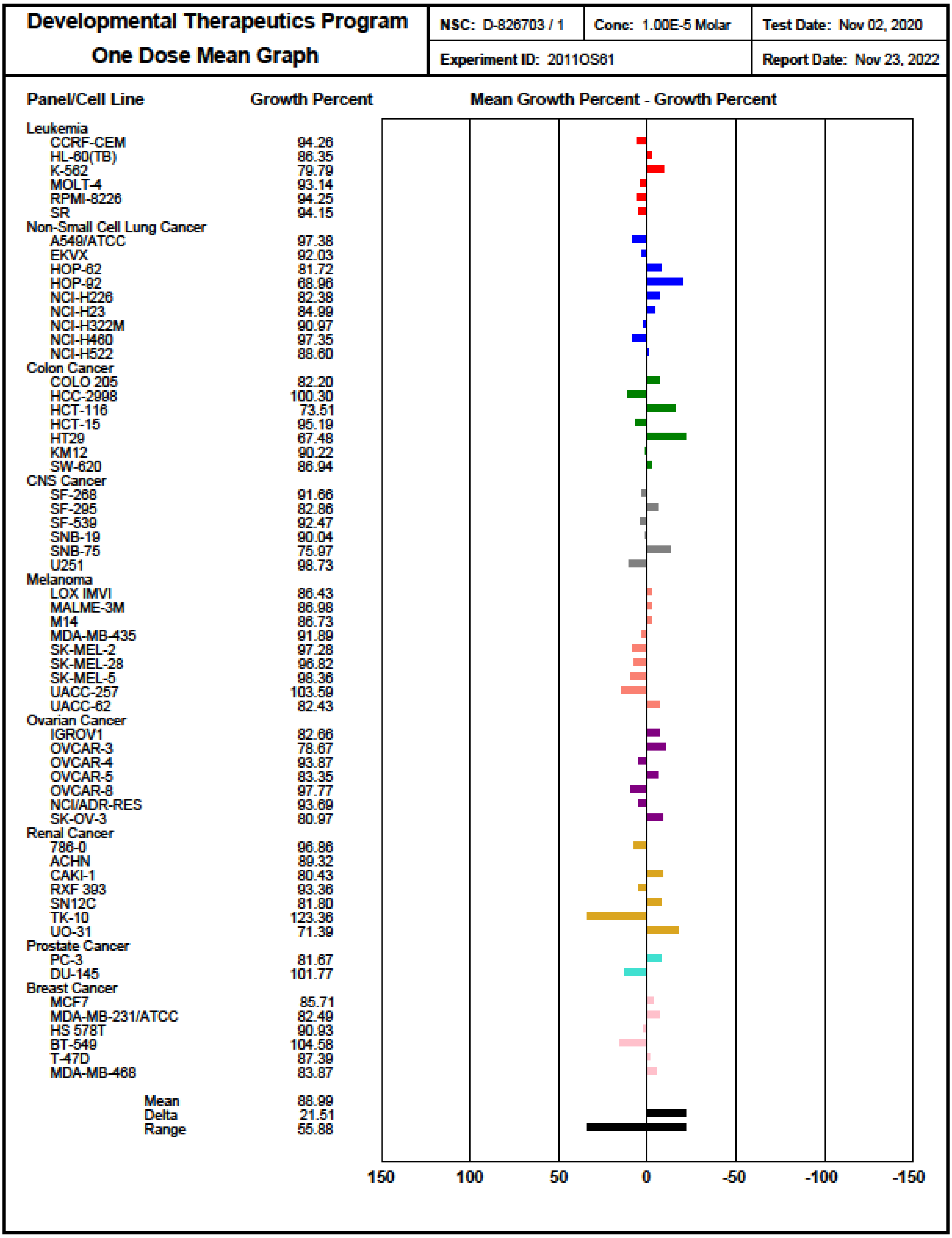

Figure S41. Results of the *in vitro* growth of cancer cell lines in the single-dose assay for **16a**

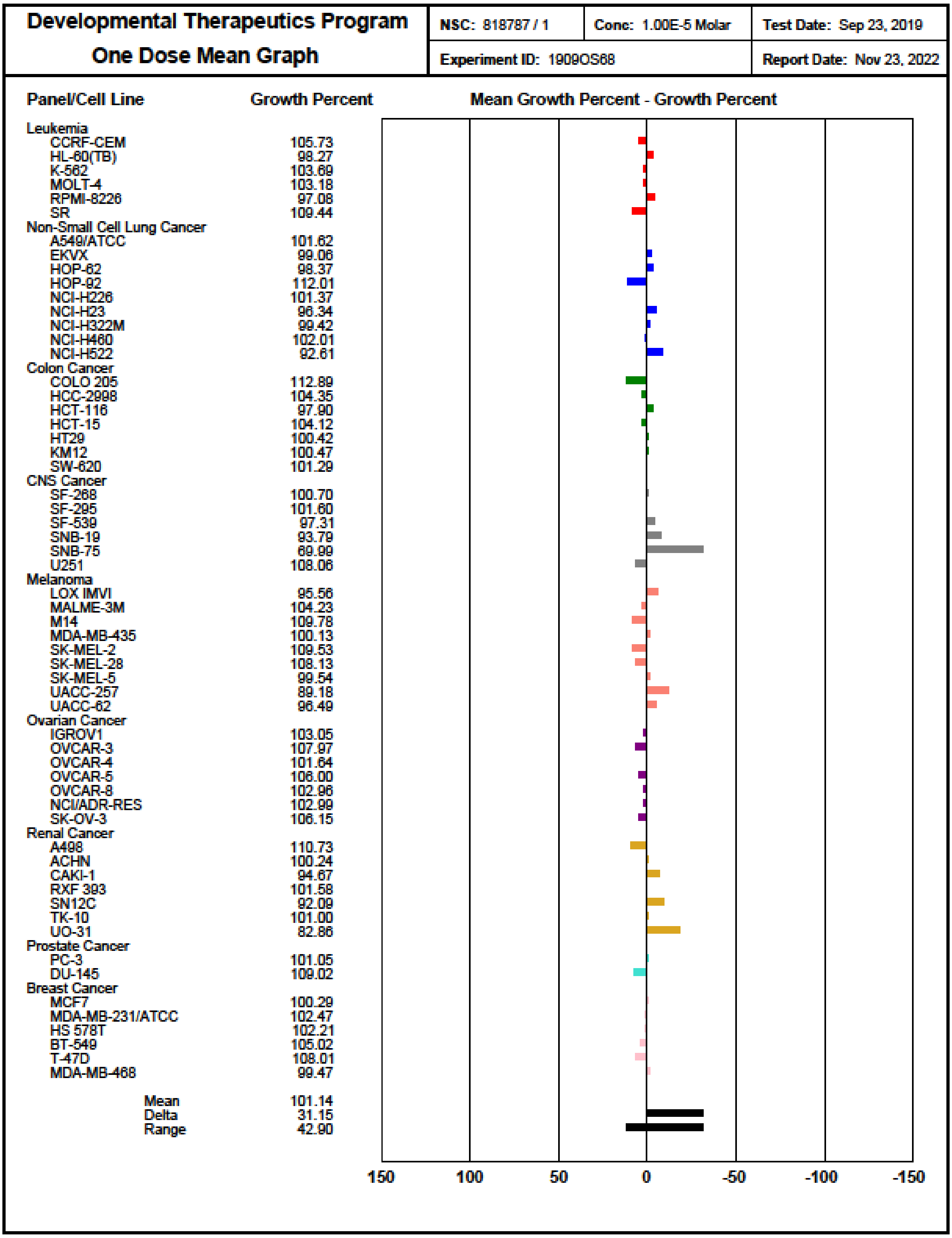

Figure S42. Results of the *in vitro* growth of cancer cell lines in the single-dose assay for **16b**

Developmental Therapeutics Program One Dose Mean Graph	NSC: 818788 / 1	Conc: 1.00E-5 Molar	Test Date: Sep 23, 2019
	Experiment ID: 1909OS68		Report Date: Nov 23, 2022

Panel/Cell Line	Growth Percent
**Leukemia**	
CCRF-CEM	101.57
HL-60(TB)	103.29
K-562	98.71
MOLT-4	103.97
RPMI-8226	108.49
SR	115.71
**Non-Small Cell Lung Cancer**	
A549/ATCC	77.88
EKVX	94.81
HOP-62	70.72
HOP-92	111.32
NCI-H226	91.85
NCI-H23	81.46
NCI-H322M	91.10
NCI-H460	100.54
NCI-H522	83.28
**Colon Cancer**	
COLO 205	114.60
HCC-2998	107.81
HCT-116	84.01
HCT-15	100.47
HT29	100.85
KM12	99.99
SW-620	101.78
**CNS Cancer**	
SF-268	78.08
SF-295	94.93
SF-539	74.08
SNB-19	55.23
SNB-75	-7.31
U251	81.79
**Melanoma**	
LOX IMVI	88.91
MALME-3M	99.08
M14	105.47
MDA-MB-435	99.46
SK-MEL-2	102.42
SK-MEL-28	106.93
SK-MEL-5	100.11
UACC-257	93.37
UACC-62	91.13
**Ovarian Cancer**	
IGROV1	101.54
OVCAR-3	112.03
OVCAR-4	88.81
OVCAR-5	98.04
OVCAR-8	89.86
NCI/ADR-RES	92.84
SK-OV-3	98.20
**Renal Cancer**	
A498	89.33
ACHN	84.24
CAKI-1	84.52
RXF 393	61.54
SN12C	81.21
TK-10	99.77
UO-31	95.31
**Prostate Cancer**	
PC-3	99.42
DU-145	99.96
**Breast Cancer**	
MCF7	99.17
MDA-MB-231/ATCC	80.11
HS 578T	82.49
BT-549	74.68
T-47D	94.96
MDA-MB-468	74.54
Mean	91.36
Delta	98.67
Range	123.02

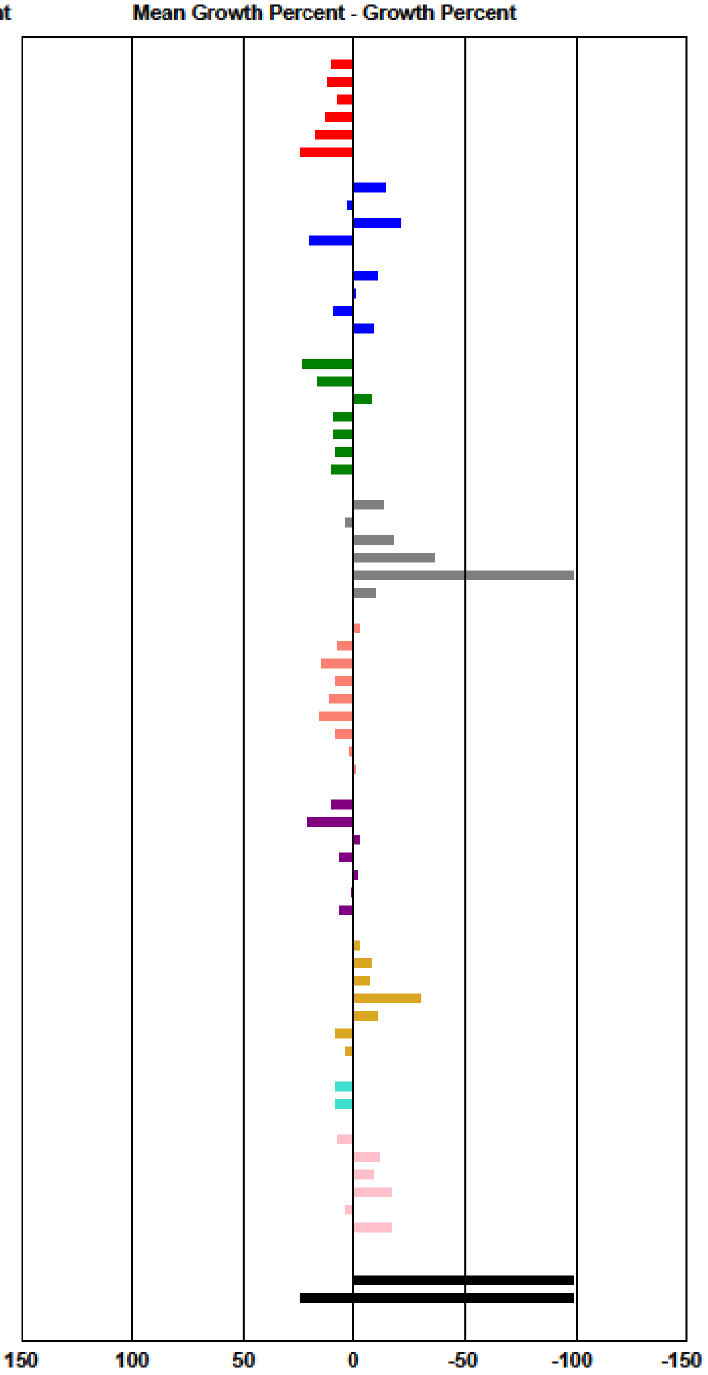

Figure S43. Results of the *in vitro* growth of cancer cell lines in the single-dose assay for **16c**

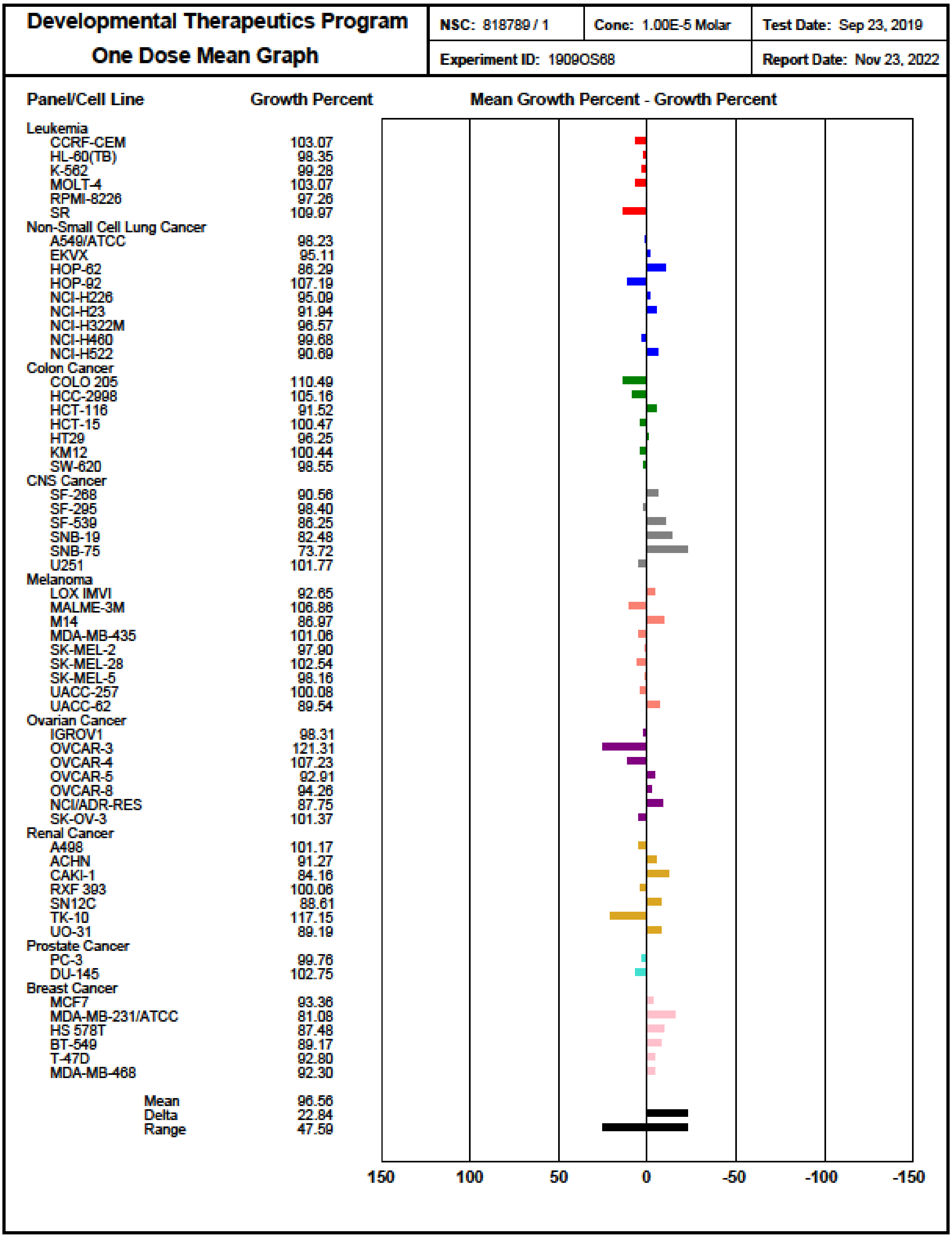

Figure S44. Results of the *in vitro* growth of cancer cell lines in the single-dose assay for **16d**

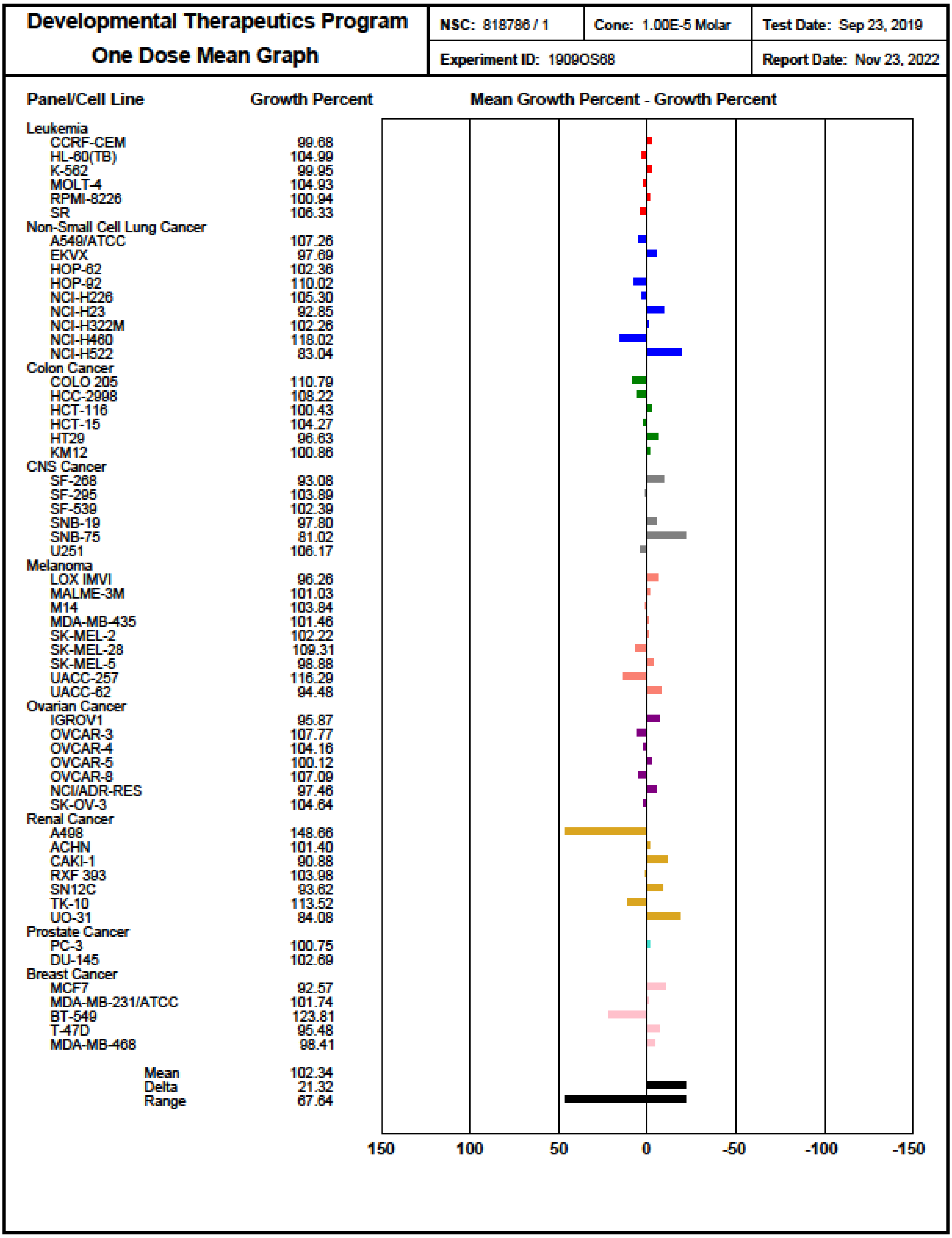

Figure S45. Results of the 5-dose *in vitro* human cancer cell growth inhibition for **10a**

National Cancer Institute Developmental Therapeutics Program In-Vitro Testing Results

NSC : 818823 / 1	Experiment ID : 2002RS18	Test Type : 08	Units : Molar
Report Date : November 23, 2022	Test Date : February 24, 2020	QNS :	MC :
COMI : R257	Stain Reagent : SRB Dual-Pass Related	SSPL : 0GLI	

			Log10 Concentration												
	Time		Mean Optical Densities					Percent Growth							
Panel/Cell Line	Zero	Ctrl	-8.0	-7.0	-6.0	-5.0	-4.0	-8.0	-7.0	-6.0	-5.0	-4.0	GI50	TGI	LC50
**Leukemia**															
CCRF-CEM	0.478	2.462	2.337	2.093	0.820	0.766	0.801	94	81	17	15	16	3.09E-7	> 1.00E-4	> 1.00E-4
HL-60(TB)	0.735	3.138	3.134	2.685	0.781	0.731	0.765	100	81	2	0	1	2.47E-7	.	> 1.00E-4
K-562	0.199	2.083	2.093	0.763	0.440	0.372	0.491	101	30	13	9	15	5.20E-8	> 1.00E-4	> 1.00E-4
MOLT-4	0.531	2.794	2.706	2.636	1.114	1.015	1.221	96	93	26	21	30	4.36E-7	> 1.00E-4	> 1.00E-4
RPMI-8226	0.853	2.698	2.619	2.544	1.250	1.153	1.170	96	92	22	16	17	3.92E-7	> 1.00E-4	> 1.00E-4
**Non-Small Cell Lung Cancer**															
A549/ATCC	0.348	2.021	1.998	1.509	0.679	0.468	0.646	99	69	20	7	18	2.46E-7	> 1.00E-4	> 1.00E-4
EKVX	0.635	1.819	1.738	1.523	1.019	0.953	0.993	93	75	32	27	30	3.86E-7	> 1.00E-4	> 1.00E-4
HOP-62	0.799	2.152	2.016	1.756	1.293	1.091	1.021	90	71	37	22	16	4.04E-7	> 1.00E-4	> 1.00E-4
HOP-92	1.161	1.843	1.708	1.557	1.594	1.374	1.345	80	58	64	31	27	2.62E-6	> 1.00E-4	> 1.00E-4
NCI-H226	0.901	2.219	2.158	2.096	1.705	1.258	1.493	95	91	61	27	45	2.11E-6	> 1.00E-4	> 1.00E-4
NCI-H23	0.626	2.051	1.959	1.790	1.032	0.836	1.000	94	82	28	15	26	3.94E-7	> 1.00E-4	> 1.00E-4
NCI-H322M	0.747	2.105	2.031	1.924	1.340	1.230	1.291	95	87	44	36	40	7.11E-7	> 1.00E-4	> 1.00E-4
NCI-H460	0.404	3.248	3.261	3.094	0.713	0.570	0.748	100	95	11	6	12	3.41E-7	> 1.00E-4	> 1.00E-4
NCI-H522	1.040	2.559	2.308	1.578	1.013	0.953	0.877	83	35	-3	-8	-16	4.97E-8	8.52E-7	> 1.00E-4
**Colon Cancer**															
COLO 205	0.434	1.541	1.548	1.296	0.411	0.276	0.374	101	78	-5	-36	-14	2.16E-7	8.61E-7	> 1.00E-4
HCC-2998	0.802	2.559	2.456	2.311	1.304	1.033	1.577	94	86	29	13	44	4.23E-7	> 1.00E-4	> 1.00E-4
HCT-116	0.281	2.633	2.516	1.837	0.719	0.439	0.687	95	66	19	7	17	2.19E-7	> 1.00E-4	> 1.00E-4
HCT-15	0.357	2.235	2.188	1.280	0.721	0.599	0.747	97	49	19	13	21	9.60E-8	> 1.00E-4	> 1.00E-4
HT29	0.281	1.851	1.765	1.429	0.286	0.226	0.252	95	73	0	-20	-10	2.08E-7	1.04E-6	> 1.00E-4
KM12	0.982	3.401	3.389	2.788	1.788	1.717	1.960	99	75	33	30	40	3.95E-7	> 1.00E-4	> 1.00E-4
SW-620	0.326	2.137	2.087	1.218	0.768	0.672	0.689	97	49	24	19	20	9.64E-8	> 1.00E-4	> 1.00E-4
**CNS Cancer**															
SF-268	0.835	2.594	2.511	2.207	1.798	1.480	1.585	95	78	55	37	43	1.83E-6	> 1.00E-4	> 1.00E-4
SF-295	0.990	2.934	2.820	1.710	1.025	0.869	1.112	94	37	2	-12	6	5.93E-8	.	> 1.00E-4
SF-539	1.035	2.889	2.783	2.555	0.896	0.569	0.985	94	82	-13	-45	-5	2.16E-7	7.23E-7	> 1.00E-4
SNB-19	0.777	2.367	2.261	2.105	1.518	1.440	1.375	93	84	47	42	38	8.09E-7	> 1.00E-4	> 1.00E-4
SNB-75	1.792	2.762	2.389	2.282	1.754	1.558	1.718	62	51	-2	-13	-4	1.02E-7	9.11E-7	> 1.00E-4
**Melanoma**															
LOX IMVI	0.425	2.659	2.512	1.975	1.481	1.037	1.328	93	69	47	27	40	7.51E-7	> 1.00E-4	> 1.00E-4
MALME-3M	0.579	1.119	1.092	0.930	0.866	0.655	0.722	95	65	53	14	26	1.20E-6	> 1.00E-4	> 1.00E-4
M14	0.591	2.492	2.360	1.674	0.703	0.595	0.601	93	57	6	0	1	1.37E-7	> 1.00E-4	> 1.00E-4
MDA-MB-435	0.558	2.751	2.687	0.781	0.203	0.229	0.430	97	10	-64	-59	-23	3.48E-8	1.37E-7	.
SK-MEL-2	1.169	2.526	2.456	2.164	1.649	1.500	1.558	95	73	35	24	29	4.11E-7	> 1.00E-4	> 1.00E-4
SK-MEL-28	0.646	1.807	1.799	1.459	1.281	0.890	1.188	99	70	55	21	47	1.37E-6	> 1.00E-4	> 1.00E-4
SK-MEL-5	1.030	3.236	3.219	2.830	1.757	1.291	1.641	99	82	33	12	28	4.46E-7	> 1.00E-4	> 1.00E-4
UACC-257	0.872	2.139	2.031	1.662	1.619	1.301	1.452	91	62	59	34	46	2.27E-6	> 1.00E-4	> 1.00E-4
UACC-62	1.111	2.954	2.868	2.156	1.770	1.509	1.717	95	57	36	22	33	2.09E-7	> 1.00E-4	> 1.00E-4
**Ovarian Cancer**															
IGROV1	0.594	2.420	2.371	1.816	1.169	0.892	1.063	97	67	31	16	26	3.00E-7	> 1.00E-4	> 1.00E-4
OVCAR-3	0.638	2.016	2.083	1.232	0.470	0.309	0.486	105	43	-26	-52	-24	7.74E-8	4.18E-7	.
OVCAR-4	0.671	1.722	1.644	1.548	1.251	1.091	1.206	93	83	55	40	51	.	> 1.00E-4	> 1.00E-4
OVCAR-5	0.409	1.379	1.293	1.265	0.720	0.562	0.724	91	88	32	16	32	4.79E-7	> 1.00E-4	> 1.00E-4
OVCAR-8	0.683	2.887	2.846	2.729	1.292	1.258	1.267	98	93	28	26	26	4.54E-7	> 1.00E-4	> 1.00E-4
NCI/ADR-RES	0.554	2.180	2.131	1.423	0.631	0.648	0.645	97	53	5	6	6	1.18E-7	> 1.00E-4	> 1.00E-4
SK-OV-3	0.977	1.924	1.888	1.748	1.381	1.078	1.298	96	81	43	11	34	6.47E-7	> 1.00E-4	> 1.00E-4
**Renal Cancer**															
786-0	0.796	2.914	2.843	2.604	1.719	1.162	1.280	97	85	44	17	23	7.01E-7	> 1.00E-4	> 1.00E-4
A498	2.085	2.824	2.659	2.187	1.512	1.380	1.621	78	14	-28	-34	-22	2.71E-8	2.15E-7	> 1.00E-4
ACHN	0.541	2.346	2.281	1.849	1.495	1.027	1.297	96	72	53	27	42	1.29E-6	> 1.00E-4	> 1.00E-4
CAKI-1	1.036	2.917	2.675	1.879	1.607	1.478	1.523	87	45	30	23	26	7.54E-8	> 1.00E-4	> 1.00E-4
RXF 393	0.878	1.739	1.731	1.557	0.765	0.642	0.755	99	79	-13	-27	-14	2.06E-7	7.23E-7	> 1.00E-4
SN12C	0.847	2.981	2.991	2.806	1.921	1.635	1.905	100	92	50	37	50	1.05E-6	> 1.00E-4	> 1.00E-4
TK-10	0.885	1.793	1.669	1.647	1.435	1.180	1.286	86	84	61	32	44	2.37E-6	> 1.00E-4	> 1.00E-4
UO-31	0.764	2.041	1.840	1.587	1.316	1.226	1.247	84	64	43	36	38	4.79E-7	> 1.00E-4	> 1.00E-4
**Prostate Cancer**															
PC-3	0.549	2.321	2.260	1.676	1.095	0.956	1.011	97	64	31	23	26	2.60E-7	> 1.00E-4	> 1.00E-4
DU-145	0.448	2.003	2.070	1.873	0.619	0.535	0.579	104	92	11	6	8	3.28E-7	> 1.00E-4	> 1.00E-4
**Breast Cancer**															
MCF7	0.464	2.138	2.058	1.190	0.763	0.628	0.766	95	43	18	10	18	7.45E-8	> 1.00E-4	> 1.00E-4
MDA-MB-231/ATCC	0.726	1.874	1.879	1.800	1.341	1.102	1.279	100	94	54	33	48	1.48E-6	> 1.00E-4	> 1.00E-4
HS 578T	1.183	2.396	2.296	2.106	1.399	1.248	1.261	92	76	18	5	6	2.80E-7	> 1.00E-4	> 1.00E-4
BT-549	1.056	2.269	2.235	2.038	1.586	1.222	1.435	97	81	44	14	31	6.77E-7	> 1.00E-4	> 1.00E-4
T-47D	0.725	1.724	1.639	1.529	1.168	1.102	1.065	91	80	44	38	34	6.97E-7	> 1.00E-4	> 1.00E-4
MDA-MB-468	0.645	1.718	1.599	1.078	0.520	0.542	0.496	89	40	-19	-16	-23	6.32E-8	4.73E-7	> 1.00E-4

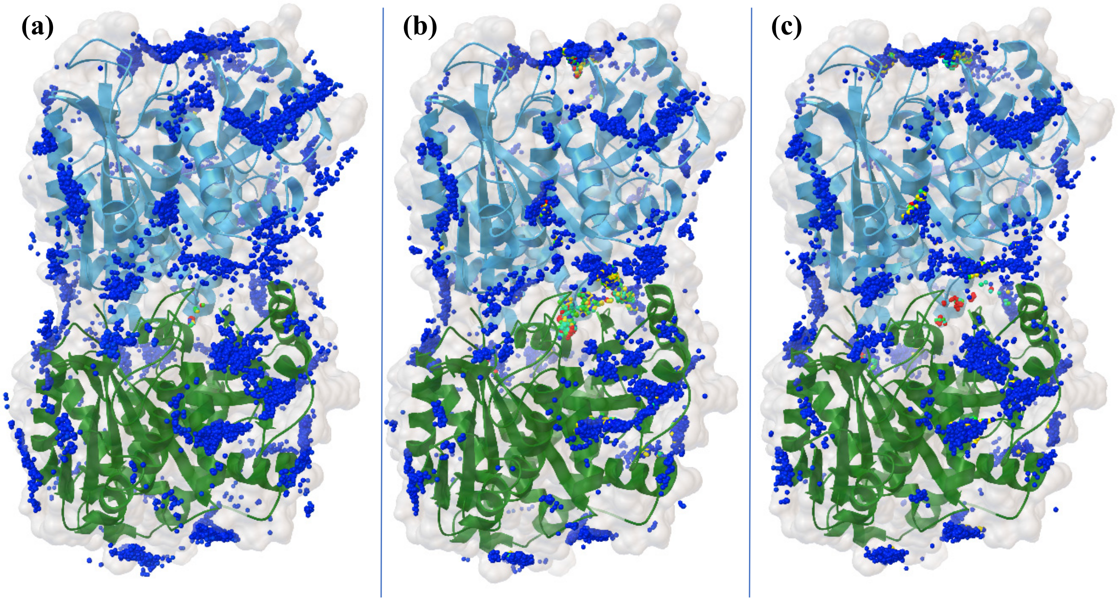

Figure S46. Conformation distribution after blind docking of compounds (a) colchicine; (b) phenstatin; (c) **10a** on the entire α,β-tubulin heterodimer (PDB ID: 4O2B); representation of all 16,000 runs of LGA for each compound; α-tubulin represented as blue ribbon; β-tubulin represented as green ribbon; α,β-tubulin heterodimer represented as transparent molecular surface; ligands represented as spheres and colored as follows: theoretical binding energy >-5.5 kcal/mol - dark blue; -6.25 kcal/mol < theoretical binding energy <-5.5 kcal/mol - orange to light blue gradient; theoretical binding energy <-6.25 kcal/mol – red

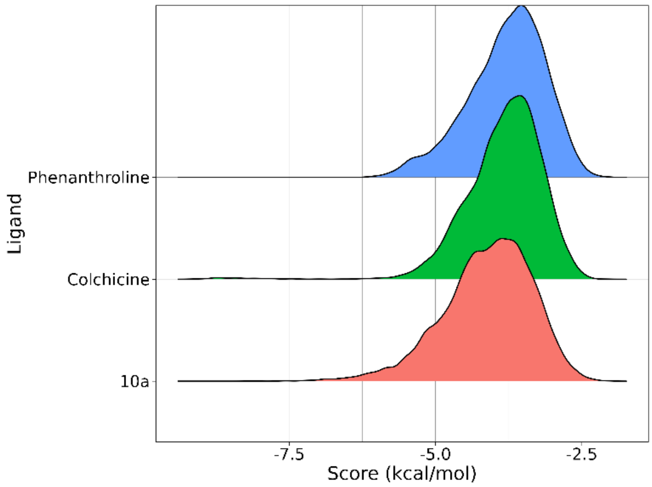

(a)

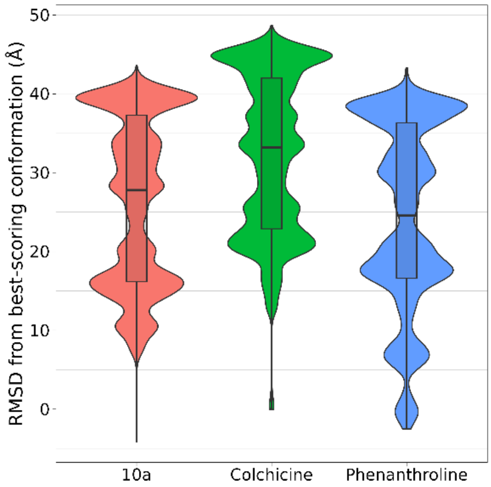

(b)

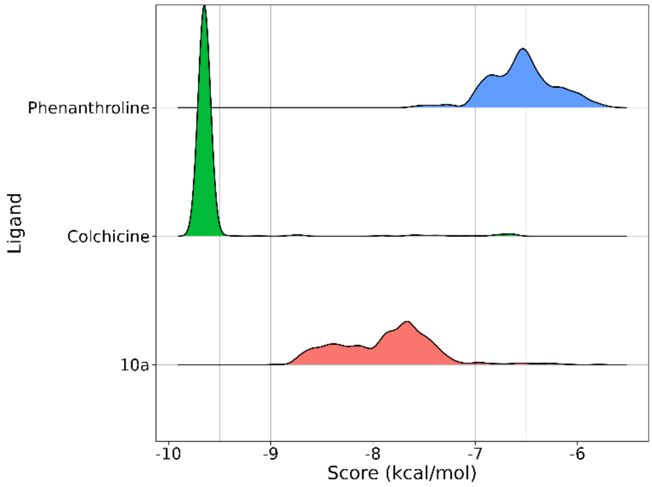

(c)

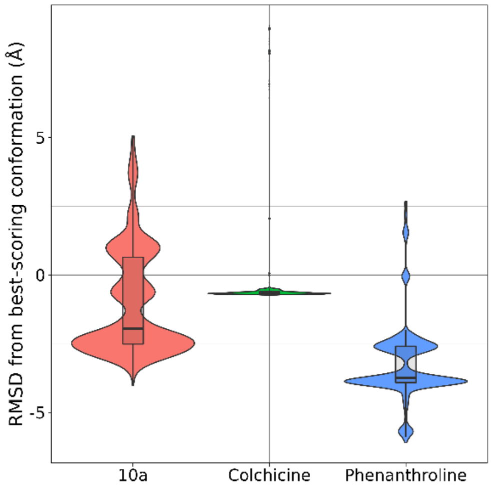

(d)

Figure S47. Binding score distribution and RMSD from lowest-scoring solution from docking experiments; **(a)** binding score distribution of global docking solutions; **(b)** RMSD from lowest-scoring conformation distribution of global docking solutions; **(c)** binding score distribution of local docking solutions; **(d)** RMSD from lowest-scoring conformation distribution of local docking solutions;

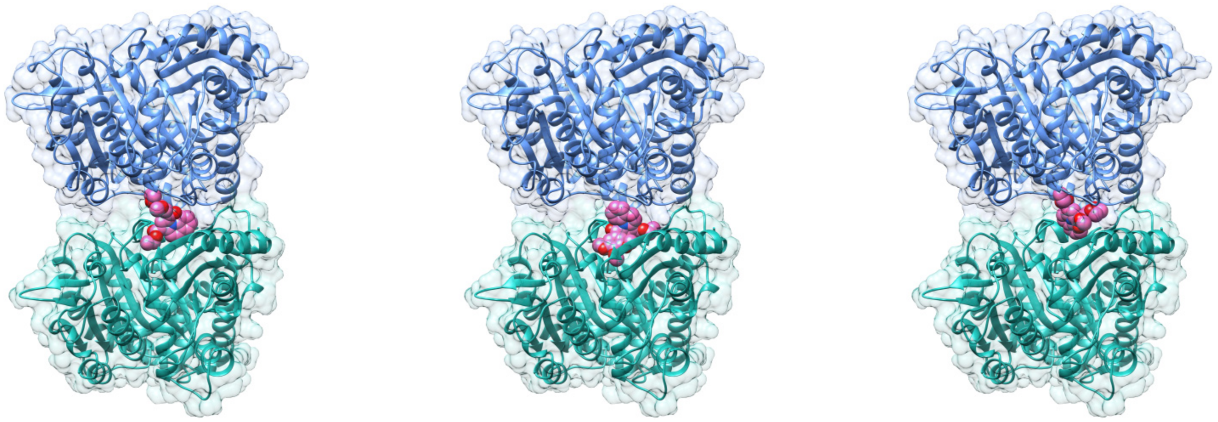

(a) **(b)** **(c)**

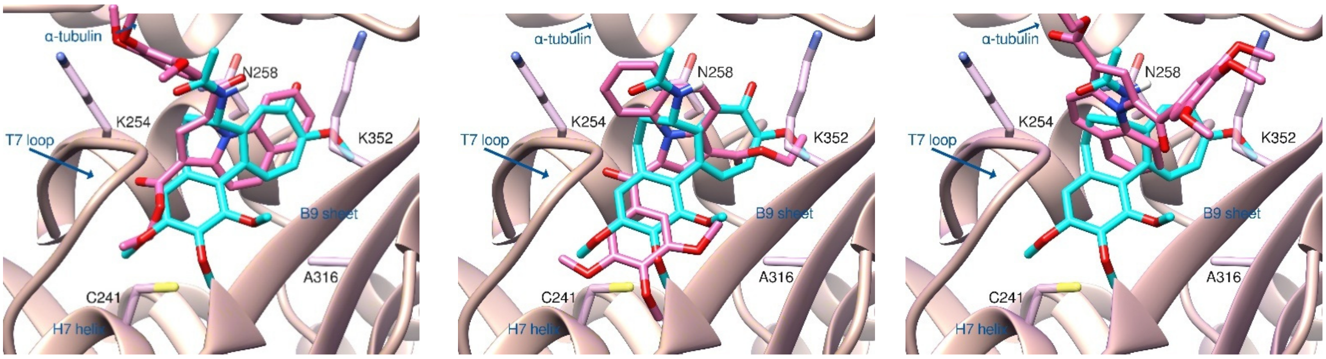

(d) **(e)** **(f)**

Figure S48. Local docking to α,β-tubulin and superimposition with colchicine for (a,d) BM I; (b,e) BM II; (c,f) BM III; for (a-c), surface and protein backbone shown in blue (α-tubulin) and green (β-tubulin); ligand shown as pink spheres; for (d-f), the α,β-tubulin heterodimer is shown as ribbons, with specific segments and amino acids labeled accordingly; **10a** shown as pink sticks; colchicine shown as cyan sticks

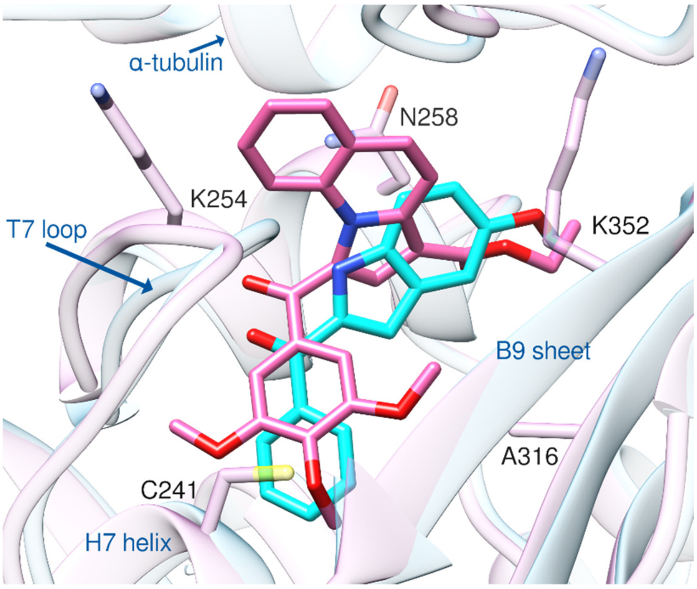

Figure S49. Superimposition of BM II of **10a** from local docking (pink) and D64131 (PDB ID 6K9V) - cyan. The α,β-tubulin heterodimer is shown as ribbons, with specific segments and amino acids being labeled accordingly.

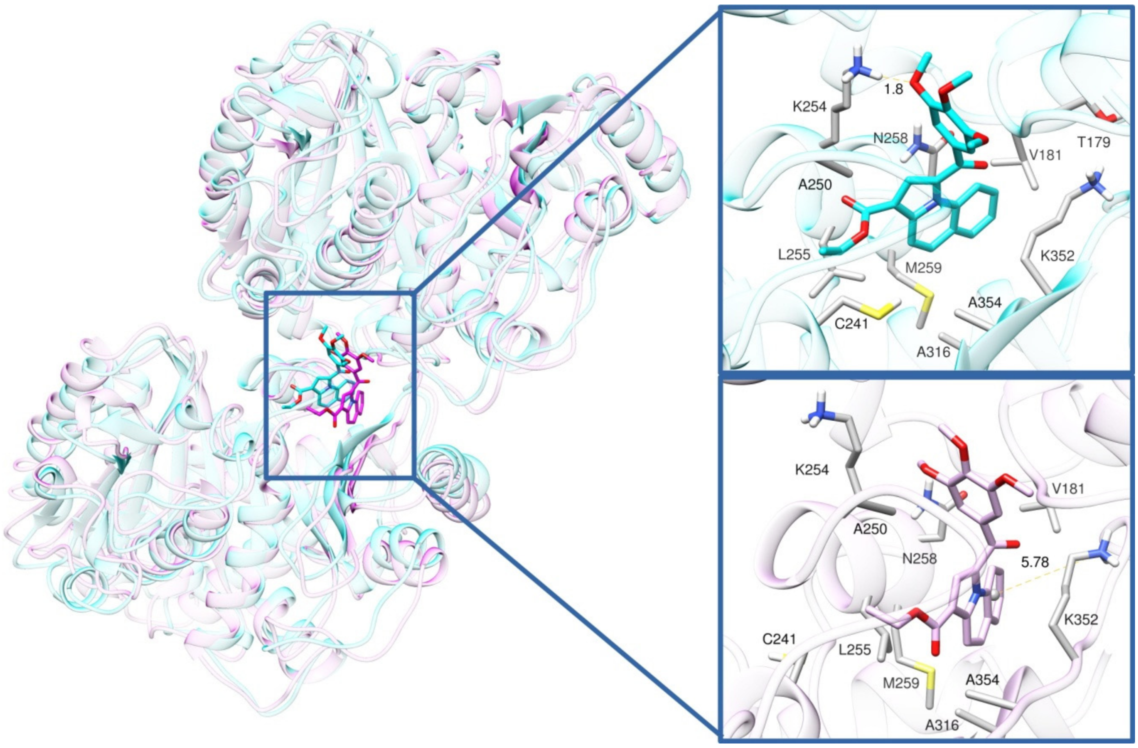

Figure S50. Superimposition of BM I of **10a** from local docking (cyan) and the last frame of the 10 ns MD simulation (pink). The α,β-tubulin heterodimer is shown as ribbons, and specific amino acids are labeled accordingly. Distances in protein-ligand H-bonds and Cation-Pi contacts are labeled.

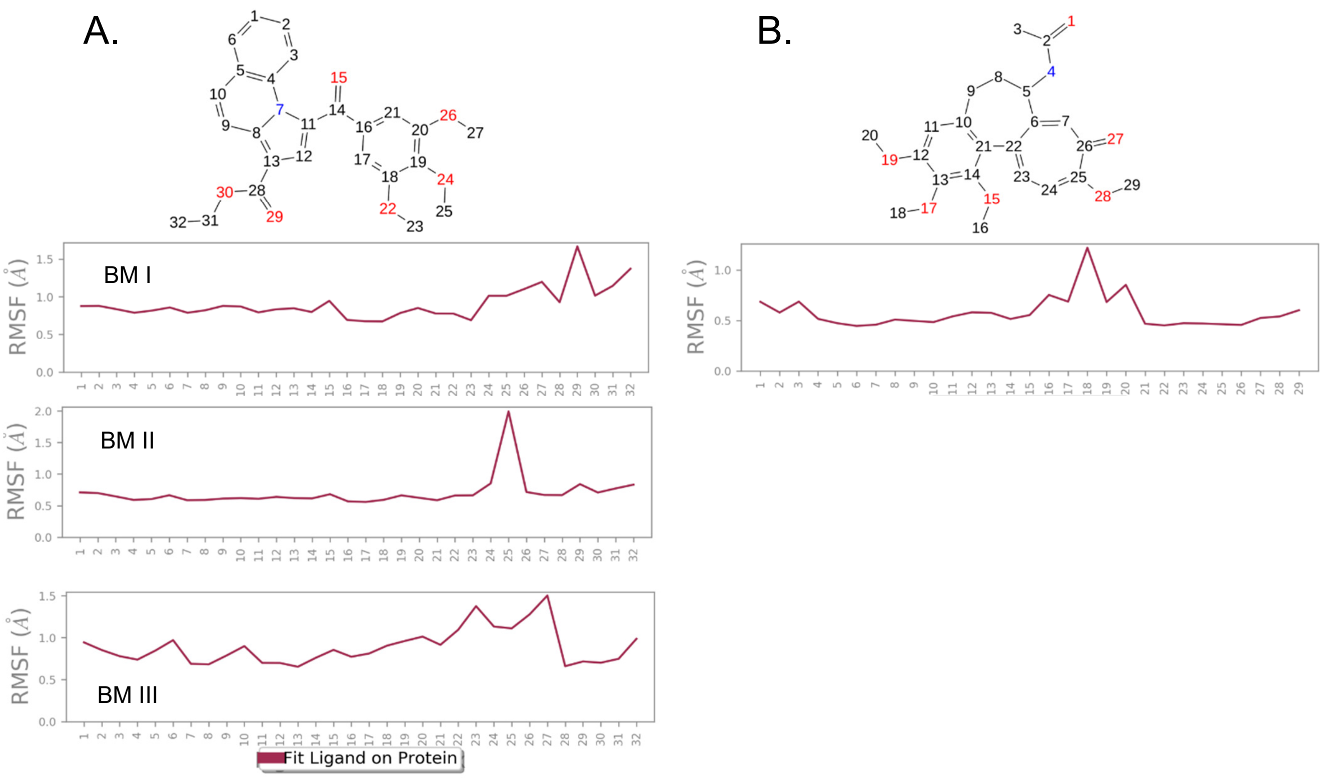

Figure S51. Ligand Root Mean Square Fluctuation (RMSF) throughout the simulations for (A) **10a** and (B) Colchicine

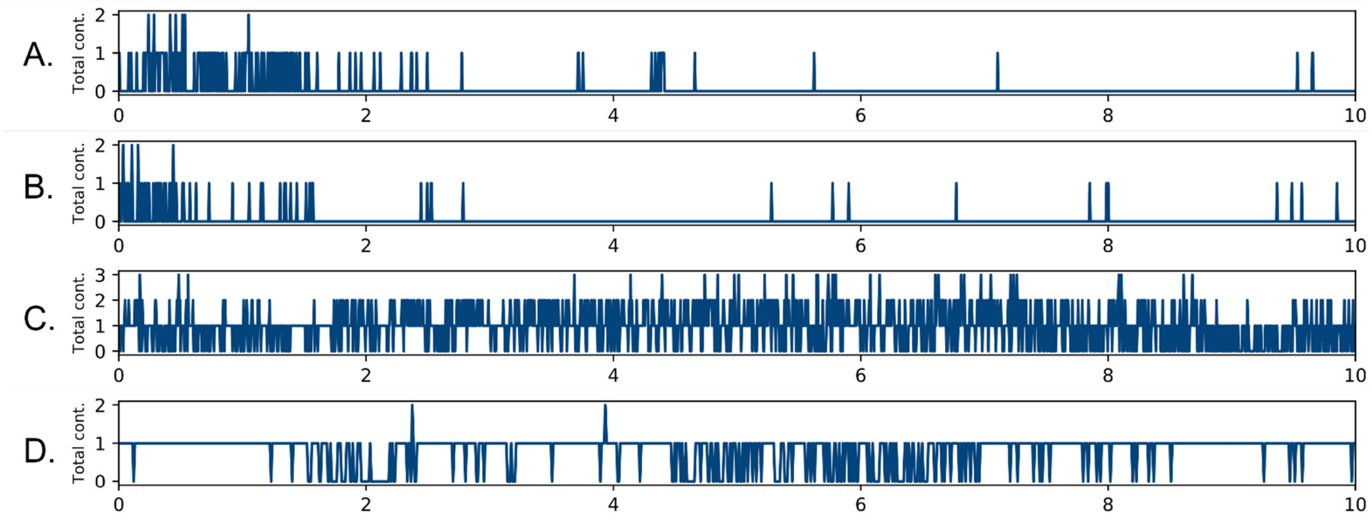

Figure S52. Timeline representation of number of H-bond interactions throughout the MD simulations for (A) BM I; (B) BM II; (C) BM III; (D) Colchicine

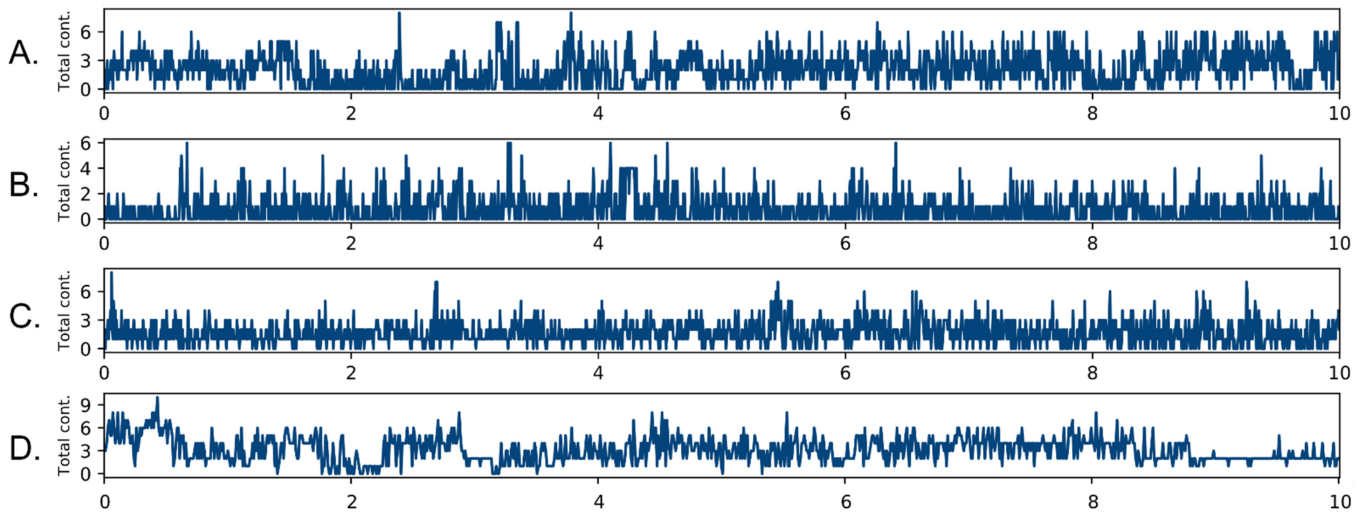

Figure S53. Timeline representation of number of polar interactions throughout the MD simulations for (A) BM I; (B) BM II; (C) BM III; (D) Colchicine

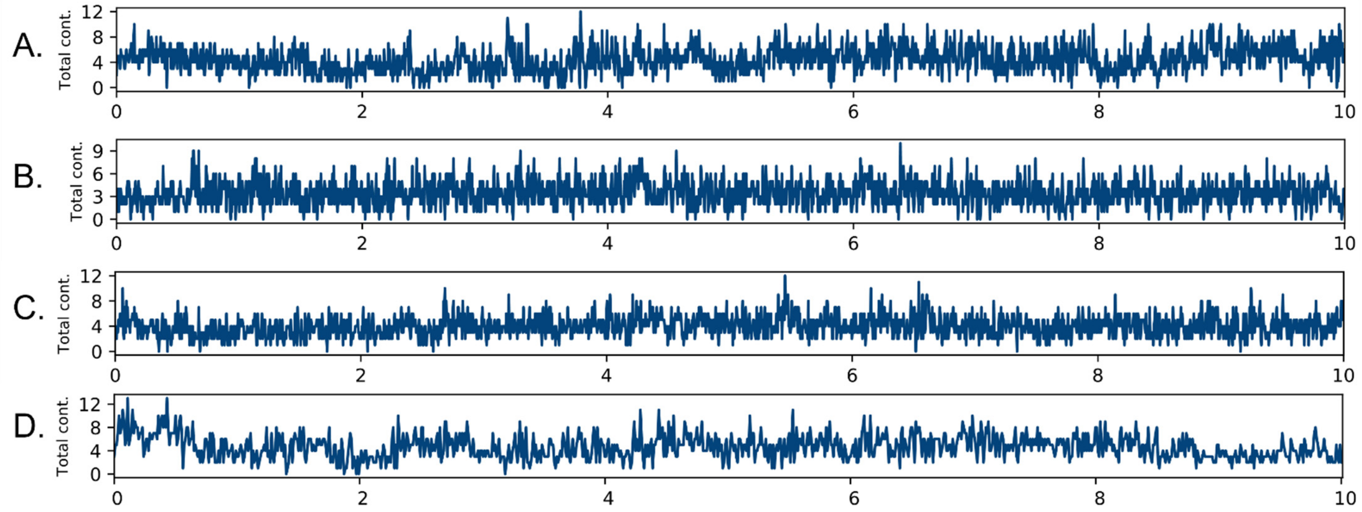

Figure S54. Timeline representation of number of all interactions throughout the MD simulations for (A) BM I; (B) BM II; (C) BM III; (D) Colchicine

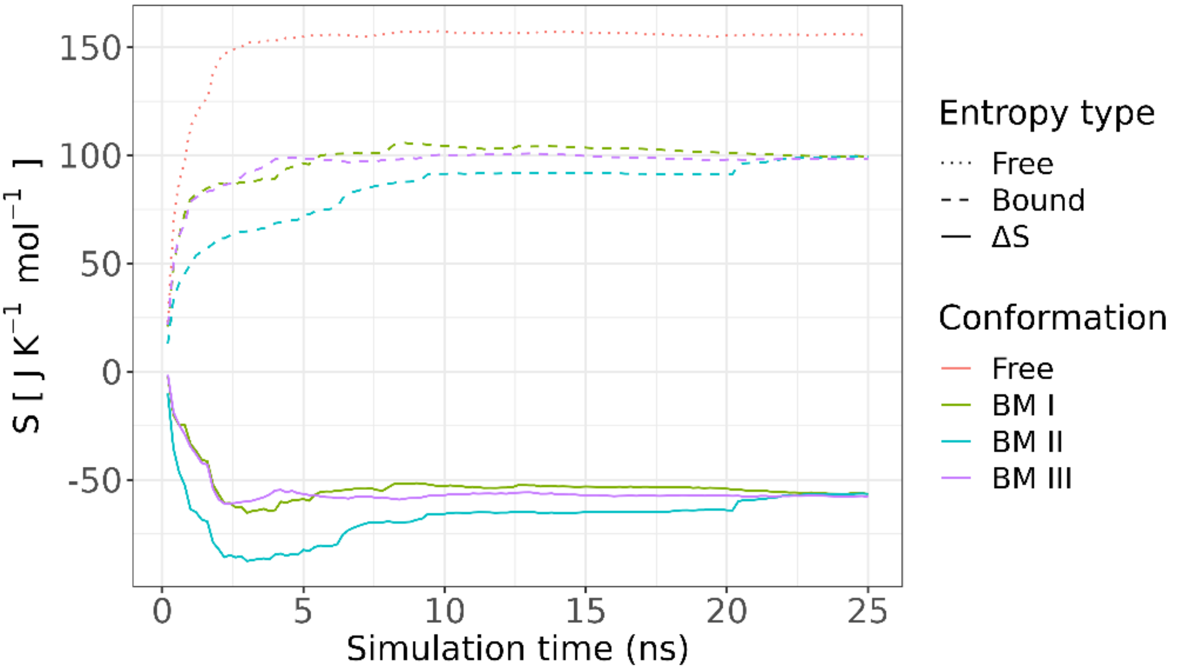

Figure S55. Configurational entropy for free **10a** (dotted line), BM I, BM II and BM III when bound to tubulin (dashed lines), and the difference between bound and free state (solid line); Lines are colored by conformation
